# Investigating mental wellbeing self-care in higher education using BERTopic modeling

**DOI:** 10.1007/s44192-025-00323-1

**Published:** 2025-11-23

**Authors:** Mahmoud Ali, Niels van Berkel, Benjamin Tag, Ville Paananen, Jonas Oppenlaender, Koji Yatani, Simo Hosio

**Affiliations:** 1 University of Oulu, Oulu, Finland; 2 Aalborg University, Aalborg, Denmark; 3 University of New South Wales, Sydney, Australia; 4 University of Tokyo, Tokyo, Japan

**Keywords:** Selfcare, Mental wellbeing, Higher education, BERTopic

## Abstract

Addressing the mental wellbeing of higher education students is urgent, given rising distress rates and significant help-seeking gaps. Students face various life challenges ranging from academic pressure and career concerns to global issues like climate change, all of which may negatively impact their mental wellbeing. While appropriate self-care can mitigate these challenges, understanding the strategies students use independently is key to developing accessible support. This article analyses contemporary triggers for mental distress and the corresponding self-care strategies adopted by students, based on data collected during the COVID-19 pandemic. We then discuss how these findings can inform the design of future digital mental wellbeing solutions. We conducted an online study with 810 participants, utilizing computational methods to analyse open-ended data. We present insights into prevalent challenges and self-care strategies, deriving direct implications for design. Finally, we discuss how technology designers can contribute to effective mental wellbeing solutions based on our findings.

## Introduction

Mental wellbeing is critical for overall health and essential for individuals to lead fulfilling lives. It encompasses emotional, psychological, and social dimensions. Mental wellbeing involves two components: hedonic wellbeing, emphasizing feeling good and functioning effectively, and eudaemonic wellbeing, focusing on optimal psychological state and personal growth [110]. Globally, mental wellbeing represents a growing public-health concern, particularly among students and younger populations, with limited access to care and unequal treatment availability. Technological and scientific therapy-based interventions, such as digital platforms and persuasive technologies, offer promising scalable strategies to address these disparities and provide effective mental health support [54]. The importance of mental wellbeing is highlighted, e.g., by research findings on how long-term attention to improving mental wellbeing is associated with an up to 8.2 times reduction in the likelihood of developing mental disorders [51, 114], a form of psychological or behavioral dysfunction that negatively impacts an individual [8, 96, 99].

Among students in higher education—universities, colleges, and other institutions offering academic certifications beyond high school—mental wellbeing is a significant public health concern [12, 29, 46]. In this student population, social pressure, increased responsibilities, demand for autonomy, and moving away from family coincide with a period of time when the brain is developing at an increasing rate, and the environment offers more opportunities for alcohol and recreational drug use [18, 26, 81]. An estimated 75% of mental health-related disorders have their onset by age 25 [12].

The term “self-care” itself is in a constant state of evolution [112]. Contemporary work reframes self-care as a multilevel construct that includes individual behaviors (e.g., sleep, exercise, mindfulness), digital and community resources (e.g., apps, online communities, institutional programs), and the sociocultural and institutional contexts that enable or constrain these practices [71, 95, 105]. While related, we distinguish self-care from adjacent concepts. *Coping* refers broadly to cognitive and behavioral strategies for managing stressors [61], which may include but are not limited to self-care. *Help-seeking* involves reaching out for professional or informal support [87], whereas self-care emphasizes actions initiated and maintained by the individual, with or without external assistance. *Digital self-help* refers specifically to the use of technology-based interventions (e.g., apps, online programs) [104], which can be understood as one subset of self-care practices.

In our work, we focus on self-care as defined by the World Health Organization (WHO): “*the ability of individuals, families, and communities to promote their own health, prevent disease, maintain health, and to cope with illness and disability with or without the support of a health or care worker*” [115]. This definition emphasises the promotion of health and coping with illness and the acknowledgement of the potential need for professional assistance, which is often crucial. Effective self-care practices have been associated with improved mental health outcomes, resilience, and an overall positive impact on the quality of life [4]. The focus on self-care, as opposed to professional help, is grounded in the understanding that individuals have the capacity to actively manage their mental wellbeing. While professional help is invaluable, self-care empowers individuals to take proactive steps in maintaining their mental health on a day-to-day basis. However, higher-education research warns against an overemphasis on individual responsibility, sometimes referred to as responsibilization, where institutional duties for student wellbeing are shifted onto students themselves [27]. This perspective highlights the need to balance self-care with institutional support systems, ensuring that wellbeing is not framed solely as a personal obligation but as a shared responsibility between students and universities.

The theoretical literature has also matured: researchers increasingly situate self-care within stress-coping and self-regulation frameworks, ecological models of wellbeing, and public-health perspectives that emphasize both preventive and promotive functions. This shift emphasizes mechanisms (e.g., how behavioral regulation, social support, and digital prompts can reduce physiological and cognitive stress responses) and boundary conditions (for whom and under what circumstances self-care is effective). Linking those theoretical accounts to higher-education contexts, recent longitudinal studies show how pandemic disruptions altered coping strategies and increased reliance on digital self-care tools, while also revealing inequities in who benefits from such tools [2, 118]. These inequities are particularly salient for international, first-generation, and low-income students, who may face additional barriers to engaging in self-care [3, 7, 49]. Equity-oriented debates in higher education emphasize that effective wellbeing strategies must account for these disparities rather than assume uniform access or benefit.

Emerging lines of work emphasize co-design with students, rigorous evaluation in real-world campus settings, and hybrid models that combine digital support with campus services [100, 104]. These developments motivate studies that examine how students choose, adapt, and sustain self-care techniques. Technology researchers and practitioners are uniquely positioned to enhance wellbeing in our increasingly digital world. Research in Human Computer Interaction for example has aimed at understanding and improving the interaction and cooperation between humans and computational tools. Given how technologies play a vital role in mental wellbeing [93, 102], our work seeks to contribute to the development of solutions that promote mental wellbeing among higher education students. Our focus is specifically on *self-care*: a way of maintaining both mental wellbeing and health. Correctly chosen self-care practices have been found to help improve students’ mental wellbeing [30, 72, 77, 117].

In this paper, we present a large-scale computational analysis of self-care practices among students, using data collected through an online questionnaire during the COVID-19 pandemic. In practice the questionnaire is part of user onboarding to a broader interactive mental wellbeing deployment, which is outside the scope of this article. Through the analysis, we answer the following research questions:

- **RQ1** How do students select mental health self-care techniques to try?
- **RQ2** How do higher education students perceive and practice self-care to address their current mental wellbeing challenges?

Answering these questions enables the identification of design considerations for developing future digital mental wellbeing solutions tailored to higher-education students. Our study revealed a range of challenges faced by this population, including emotional, financial, academic, social, and wellbeing-related issues. We found that students predominantly turn to online resources for self-care guidance, favoring techniques that are recommended by others and perceived as reliable, affordable, familiar, and easy to apply.

These findings have direct implications for multiple decision-makers. Within higher-education institutions, campus wellbeing units, student affairs offices, and counseling services can use the evidence to determine which self-care supports to promote, how best to integrate digital tools into existing services, and how to allocate limited resources. Beyond campuses, product teams developing digital mental health platforms can prioritize features that reflect students’ actual selection criteria (e.g., affordability, familiarity, ease of use). For researchers in human–computer interaction and digital health, the results highlight opportunities for co-design and evaluation of interventions that align with real-world student practices rather than idealized models of engagement.

Because the longitudinal data collection overlapped with global COVID-19 measures, the repercussions of the pandemic are clearly reflected in the dataset. Nevertheless, we argue that the findings retain relevance in post-pandemic contexts. Several patterns—including students’ reliance on online resources for guidance, their preference for affordable and easy-to-apply techniques, and the importance of peer or community recommendations—are likely to persist and reflect broader trends in digital help-seeking and self-care among young adults. The findings indicate a demand for intuitive, user-friendly platforms that support students’ mental wellbeing and integrate personalized self-care techniques tailored to the individual challenges they face. Finally, in line with open science practices and to support the broader research community, we have released the trained BERTopic models as open source.^1^  

## Related work

Self-care lacks a universally agreed-upon definition but broadly refers to individuals finding their own methods for fostering their wellbeing. Godfrey et al. [39] surveyed and presented 139 different definitions of self-care from 1976 to 2009, categorizing them based on their emphasis on one of seven key components: health, illness or disability, general outcomes, self-care performer, the action of self-care, healthcare professional, or the healthcare system. For example, one definition of self-care is “the ability to care for oneself through awareness, self-control, and self-reliance to achieve, maintain, or promote optimal health and wellbeing” [67]. Another definition is “taking the time to do things that help you live well and improve both your physical health and mental health” [75]. Nunes et al. [74] explored the intersection of HCI with self-care technologies, identifying key trends, such as the rise of personalized self-care apps and the integration of AI. The paper highlights significant challenges, including privacy concerns and accessibility issues, while outlining opportunities for enhancing user engagement and fostering interdisciplinary collaboration. Next, we provide context for our work through literature on mental wellbeing in higher education and how technologies can help analyse data or contribute solutions in this context.

### Mental wellbeing in higher education

While clinical resources, such as counselling services, play a significant role in addressing mental health concerns among higher education students, there exists a notable gap in utilization. Young adults often refrain from seeking professional treatment due to various factors, such as the perception that treatment is unnecessary, time constraints, a preference for self-management, and the fear of social stigma [20, 108]. Additionally, clinical resources may not be available in all regions (e.g., under-resourced regions). Researchers call for action to urgently gain a deeper understanding and explore potential ways to increase awareness of the importance of mental health among higher education students [26, 33, 34, 38, 45, 80]. Moreover, research emphasizes the critical impact of students’ mental wellbeing on their academic achievements [19, 32, 62]. Research has also demonstrated that the use of online mental health support varies across cultures [82].

Park et al. [79] conducted comprehensive interviews with 19 university students to understand how they seek and receive support to maintain their mental wellbeing. The study identified three needs: providing help that aligns with the severity of the problem, building relationships with potential support givers, and negotiating the tension between the need to disclose and the associated stigma. Similarly, Larcombe et al. [59] conducted a large-scale survey involving over 5000 university students, focusing on indicators of depression, anxiety, and stress. The results confirm that the mental wellbeing of students is a serious public health issue with 25% of the participants experienced very high levels of psychological distress. The study advocates for educational and health policies to recognize and address the high rates of student psychological distress. Compared to the works by Park et al. and Larcombe et al., our study employs computational analysis of data related to mental distress in higher education and provides design guidelines for addressing these challenges.

Universities play a crucial role in guiding and providing the mental health support students require [7, 13, 116]. Consequently, counselling services must be equipped to address the increasing number and severity of students’ mental wellbeing issues [11]. Research has indicated that under-resourced education systems contribute to heightened pressure among students, compromising their mental wellbeing [103]. These studies emphasize the challenges surrounding mental wellbeing in higher education while also highlighting potential solutions to address these issues.

### Computational methods for mental health insights

Computational analysis of user data from social media platforms offers researchers a rich set of data to explore and analyse a multitude of topics, including human behavior, communication patterns, and societal events. Different platforms offer different media types to analyse and in different scales. Beyond analyses of digital traces on social media, Kolenik [52] highlight methods for smartphone- and IoT-enabled assessment and just-in-time interventions, situating computational mental health research within the broader landscape of mobile sensing and digital delivery. Luo et al. [63] employed a convolutional neural networks (CNN)-based classifier to analyse 716,899 public tweets, identifying patterns related to suicidal ideation. This study revealed 13 key factors that can inform the development of effective suicide prevention strategies. Similarly, Chancellor et al. [15] analyzes 26 million Instagram posts to assess mental illness severity (MIS) in pro-eating disorder (pro-ED) communities. The study identifies a concerning rise in severe content and demonstrates that past MIS can predict future risks with high accuracy, offering insights for mental health interventions. The study employs a hybrid approach combining Latent Dirichlet Allocation (LDA) for topic modelling with human annotations. In contrast, we use BERTopic for computational analysis, a method shown to yield better topic modeling results compared to LDA [1, 28, 44].

Forums where users engage in extended discussions and can write longer-format text serve as valuable sources of information. In this context, Bagroy et al. [6] analysed student-contributed data on Reddit using a transfer learning based classification approach to evaluate the mental health status of over 100 university campuses. Moreover, MacLean et al. [65] conducted a text-driven analysis of posts on an online addiction recovery forum. Through linguistic features, the authors sought to identify a user’s addiction phase, relapse, or whether they are recovering. Gauthier et al. [36] conducted a thematic analysis of two recovery communities on Reddit using machine-generated topic models. In addition to providing insights into the workings of these communities, the authors reflect on the use of computational techniques to study communities at scale. Compared to those studies, our approach provides more nuanced insights into the mental wellbeing of higher education communities.

### Designing for mental wellbeing

Numerous studies investigated the utilization of digital tools designed to support self-care and enhance overall wellbeing. Moilanen et al. [70] compared the use of a ‘traditional’ web interface and a chatbot in supporting users to access crowdsourced self-care techniques. While participants report a lower trust for the chatbot-version in terms of security and integrity, the authors suggest chatbots may be a possible solution to overcome the resource crisis in healthcare and help users make sense of the extensive resources available online. A similar study [50] demonstrated that a self-guided mental healthcare system with a chatbot yields higher stress reduction and greater continuity of use compared to one without a chatbot. Complementing this, Kolenik et al. [55] introduced a computational psychotherapy system that combines predictive modeling, behavior change mechanisms, and theory of mind simulation in a conversational agent. Their evaluation showed interventional effects in reducing stress and anxiety. Kornfield et al. [56] presented a user-centred approach to the design of a mental health support tool. Following an online discussion group and design workshops with young adults with depression or anxiety symptoms, the authors highlight specific aspects of an automated text messaging support tool. Participants stress the importance of enabling variable levels of engagement based on user preference, availability, and mental state, as well as enabling further personalisation of the content offered by the tool. Pretorius et al. [84] conducted a study to identify key design factors for online mental health resources that can facilitate young people’s help-seeking behavior. The authors used a large online survey to gather data and developed personas based on the results. Subsequently, the study identified four key design considerations: connectedness, accessible information, personalisation, and immediacy. Lattie et al. [60] conducted a study with college students and counseling centre staff aimed at identifying the needs for digital mental health tools. The findings proposed considerations for designing future tools, emphasizing the significant role of social factors. Beyond these design-oriented perspectives, a technical review of intelligent cognitive assistants (ICAs) for mental health [53] provides a complementary view by classifying current systems according to their technological underpinnings. The review highlights how user models, classification-based assessment, personalized interventions, and dialogue tree conversational models have been employed in ICAs to support stress, anxiety, and depression (SAD).

Docherty and Biega [25] draw attention to ‘digital wellbeing’, highlighting the responsibility of HCI researchers and practitioners to consider the impact of technology on mental wellbeing. They furthermore stress that current work on digital wellbeing focuses on user engagements rather than considering the full complex context (e.g., social, cultural) that impacts users’ wellbeing. Complementing this perspective, Zhang et al. [119] interviewed 32 people with symptoms of depression or anxiety to understand their usage of mental wellbeing applications. Their results offer implications for increasing user autonomy through customisability without overburdening end-users. Sien et al. [89] engaged 19 participants, including international students and mental health professionals, in co-design sessions to develop technologies addressing the mental health needs of these students. The findings highlighted the importance of several factors for the design of mental health technologies: user preferences, helpfulness, comfort, trust, professional support, destigmatizing social support, and user agency. Our results align with many of the findings discussed in this section, but we offer more detailed guidelines derived from our computational analysis.

## Study design

Our study employs a computational analysis approach using BERTopic [42], assisted by additional manual qualitative analysis, as topic modeling may not generate the most accurate results [17]. Through this approach, we derive insights from a dataset consisting of open-ended questionnaire data collected from an interactive self-care discovery tool online. We adopt the WHO’s definition of self-care [115] and draw further inspiration from e.g., [10, 39], positioning it as an intentional activity toward taking care of oneself. Thus, for instance, simply having a great network of friends is not self-care, even if it has positive effects on mental health. On the other hand, actively cultivating that network is, as it is a deliberate action.

### Apparatus

The data analysed in this paper originate from a participant background questionnaire that is part of a large interactive online self-care deployment. It is operated and maintained by the University of Oulu. We note that the deployment itself is not the focus of this paper. In this paper, we present an in-depth analysis of the *background questionnaire* data. The data are intended to provide a broad-ranging exploration into the general mental wellbeing and its self-care among the higher education community, which is also useful for a discussion about future technology opportunities.

### Data collection through an online questionnaire

**Fig. 1:**
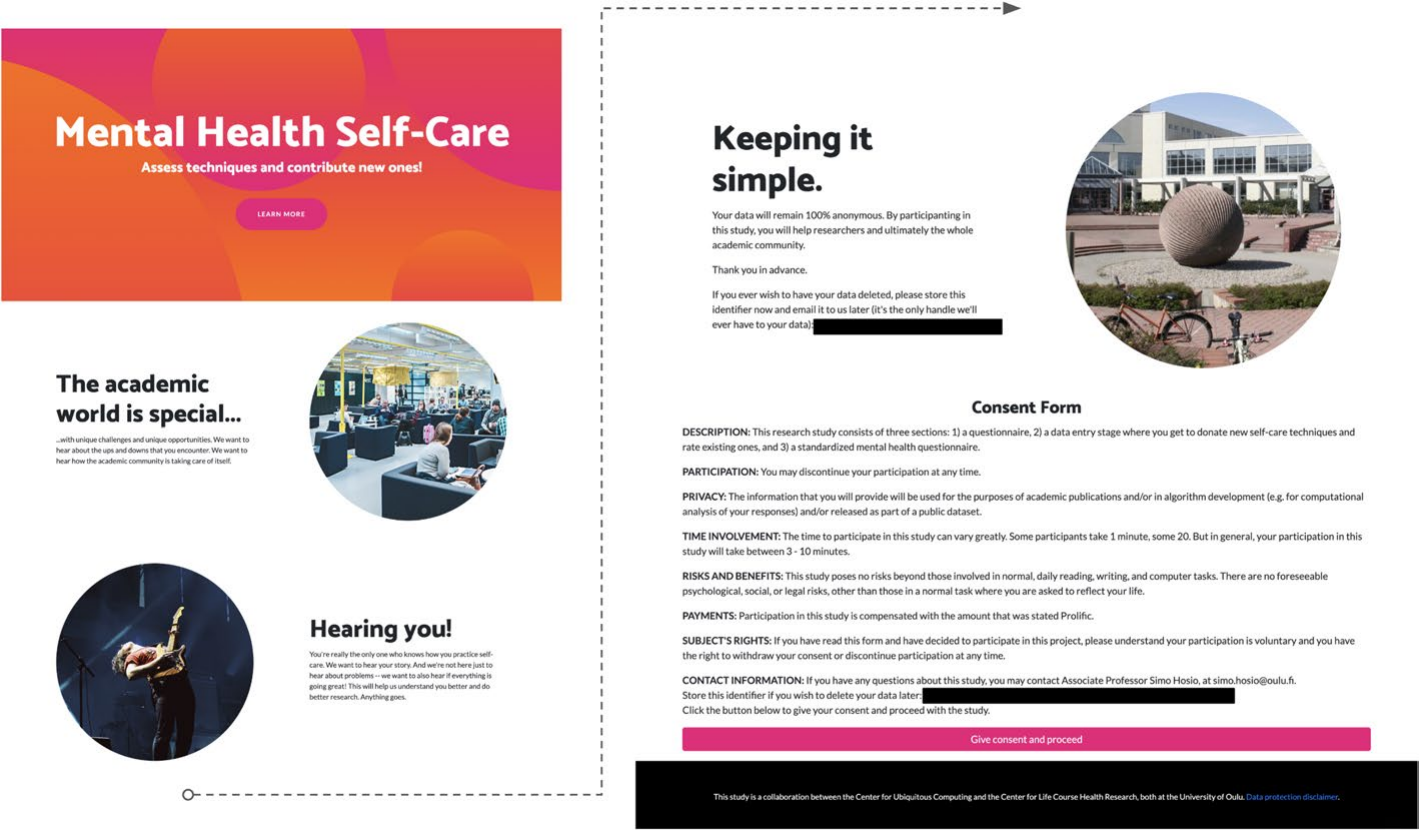
A screenshot of the full front page the participants saw before starting the questionnaire

On the landing page (Fig. 1), participants were provided with a detailed explanation of the study and a consent form, which they had to agree to before accessing the questionnaire. The consent form includes details about upcoming tasks, privacy information, estimated participation time, and information on how the data will be used in academic publications, open datasets and in algorithm/model development. In addition, the consent form outlines the user’s rights and includes contact information of the principal investigator in case of facing problems or if the participant wishes to withdraw his consent or data. We opted for a questionnaire over interviews or focus groups to reach a large and diverse participant pool efficiently, providing a breadth of perspectives essential for a nuanced exploration of mental wellbeing. Additionally, the anonymity offered by the questionnaire encourages more candid responses on sensitive topics, promoting honesty and minimizing social desirability bias, which is particularly important given the sensitive nature of the study’s subject matter.

The questionnaire was designed in collaboration with two specialised psychiatrists from Oulu University Hospital with extensive clinical and scientific experience. Together, we developed questions that would yield insights for future technology developers while remaining consistent with a realistic initial consultation with a psychiatrist. To this end, the following four questions were identified to generally capture much of the information that our expert collaborators are interested in during in-person client consultations, while also capturing novel data about self-care techniques in a non-intrusive way. All questions were optional, ensuring participants felt no pressure to answer questions they might not feel comfortable addressing in an in-person consultation.

1. If you were to find and start practising a mental health self-care technique, i.e. something that you can do on your own and without the need of an expert, how would you go about finding one? Where would you go looking for it? Even if you don’t need one now, just think about it: How would you start finding one?
2. What would affect your choice? Think of anything and everything that you might consider when choosing ‘what to try’ as a self-care technique?
3. What things in life, if anything, have ever troubled concerning your mental health?
4. Now consider the things you listed for the previous question... what do you think could help you? How could someone or something help you with the things? Again, no wrong answers here. Just think and tell us freely.

The questions were presented in a fixed order to maintain a natural conversational flow and align with the progression typically used in an in-person consultation. We acknowledge that a fixed order may introduce potential order or priming effects—for example, thinking about finding a self-care technique in Q1 could influence how participants respond to considerations in Q2. The questions align with our definition of self-care by explicitly asking about how people would find something specific to do, or how would they make choices. These questions in this order allow for a conversational and stress-free exploration of the patient’s current situation while enabling reflection on personal self-care strategies. Our aim here was to obtain a rich understanding of one’s current troubles and potential mitigation strategies, leading to a dataset that is useful for a range of stakeholders, including technology researchers. Additionally, the questionnaire included basic demographic information (age, gender, and education) as well as a Likert-style item on the participant’s overall current self-rated mental health, rated on a scale from 1 (not at all well) to 7 (extremely well). Participants were also instructed to complete an optional eight-item Patient Health Questionnaire (PHQ-8), a validated tool for assessing the severity of depressive symptoms [57]. Each of the eight items is scored from 0 (“not at all”) to 3 (“nearly every day”), yielding a total score from 0 to 24, with higher scores indicating greater symptom severity. For analyses linking PHQ-8 scores to topic clusters, we grouped scores into commonly accepted depression categories: 0–4 (none/minimal), 5–9 (mild), 10–14 (moderate), 15–19 (moderately severe), and 20–24 (severe). Participants with incomplete PHQ-8 responses were excluded from these analyses.

### Ethical considerations

Through a consultation with our institutional review boards (IRB), our study was exempt from a formal approval process. IRB approval in our institution is required for research that deals with interventions, typically randomized controlled trials in the field of medicine. Our study (and the online system) is considered as equivalent to any online questionnaires or user-generated content platforms. Yet, considering the nature of the data, we ensured each participant was only identifiable through a pseudonymous identifier in the data, which was made known to the participants already in the consent form. They were offered the right to remove their data at all times while still getting compensated for participation. No participants used this option. The survey questions did not specifically probe suicidality or acute trauma, and no support resources were provided at survey completion. We acknowledge that including such resources is a recommended practice in mental health research and will incorporate this in future studies.

### Analysis methods

Prior to analysing the data, we conducted a manual review of the responses to ensure they contain no contact information. Next, we carried out a data cleaning step involving the removal of duplicate and invalid entries, i.e. entries with a missing ID, an empty age field, or unanswered questions. We then used BERTopic [42] to extract and analyse the main topics in the participants’ responses. Topic modeling is an exploratory method that generates interpretable clusters of text but does not, in itself, guarantee definitive categorization. Thus, our findings should be viewed as indicative patterns rather than conclusive representations. BERTopic is a topic modelling technique that uses BERT (Bidirectional Encoder Representations from Transformers) [23], a deep learning-based natural language processing model to identify topics within a corpus of text data. Unlike traditional topic modelling techniques, such as LDA [9], which rely on statistical methods, BERTopic uses a clustering algorithm (HDBSCAN [68]) to group similar documents together and then extracts topics based on the most representative documents within each cluster. Within the context of BERT, a document refers to a single text entry in the model’s training data, even if it contains only one word. While BERTopic does not generate a single concise label for a topic, the most representative terms it provides can give insight into each topic’s focus. In practice, assigning a descriptive concise label often requires manual work. For the representation_model parameter, we used a MaximalMarginalRelevance model with the diversity parameter set to 0.2 to balance relevance and variety in the topic keywords, ensuring concise yet comprehensive topic descriptions. Although our study was conducted in English, some Finnish participants answered in Finnish. To accommodate this, we utilized the multilingual embedding model called “paraphrase-multilingual-mpnet-base-v2” [85], which offers support for over 50 languages, including Finnish. No explicit translation or language identification was applied; instead, the model encodes each response in its original language. Tokenization and stop word removal were handled via a CountVectorizer model, while other preprocessing steps, such as lemmatization, stemming, or diacritic normalization, were not applied, as BERTopic’s embeddings capture semantic meaning directly from raw text across languages.

BERTopic first encodes text documents using a pre-trained BERT model to obtain dense vector representations (embeddings). In our study, we used the multilingual model paraphrase-multilingual-mpnet-base-v2, which supports Finnish and English. These embeddings were reduced in dimensionality using UMAP [69] with default parameters (n_neighbors=15, n_components=5, metric=cosine). The reduced embeddings were clustered with HDBSCAN [68], where we tuned min_cluster_size per question and left all other parameters, including min_samples, at their defaults. Topic representations were generated with c-TF-IDF [42] and refined with a CountVectorizer limited to unigrams (ngram_range=(1,1)) and stop word removal. Since BERTopic embeddings capture contextual meaning directly from raw sentences, we did not apply additional preprocessing such as lemmatization, stemming, or diacritic normalization, and we relied on the multilingual embedding model to handle any code-switching. All other parameters were left at their defaults. For more technical details on BERTopic, refer to [42].

All analyses were conducted using Python 3.10.10 with BERTopic 0.14.1, UMAP 0.5.3, HDBSCAN 0.8.29, scikit-learn 1.2.2, pandas 1.5.3, and NumPy 1.23.5. Default parameters were used for HDBSCAN and UMAP. No explicit random seed was set. Although BERTopic training can yield slightly different results across runs due to the stochastic nature of UMAP initialization and HDBSCAN clustering, we saved the trained BERTopic models to ensure full reproducibility. Analyses were performed on a laptop with an Intel i9-11950H CPU, 32 GB RAM, and NVIDIA RTX A3000 GPU with 6 GB memory.

**Fig. 2:**
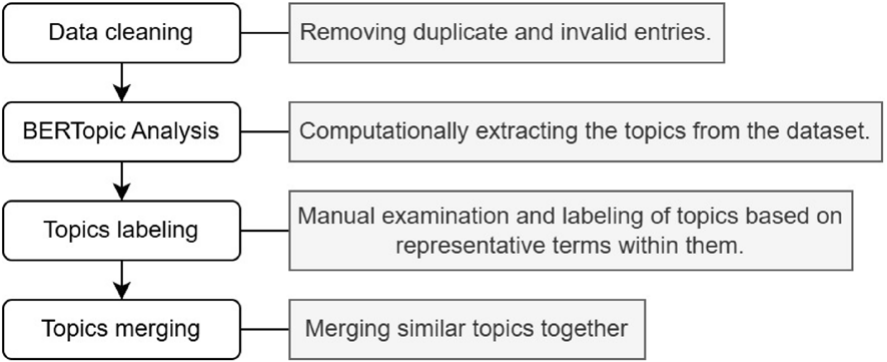
Summary of analysis methods

After clustering the documents from each of the four questions into topics using BERTopic, we reviewed the top keywords for each topic and manually assigned descriptive labels for each topic. Figure 2 provides a summary of the analysis workflow. After removing duplicate inputs, the document counts for each question were $Q1=748$, $Q2=774$, $Q3=774$, and $Q4=771$. The initial topics generated for each question contained 20–50% outliers (Table 1). Outliers are documents that do not fit well into any of the identified topics. To avoid eliminating a significant portion of the data, we applied BERTopic’s reduce_outliers() function, which reduces outliers by merging them with the most similar topic, following a chosen reduction strategy [43]. We used the default parameters (strategy="distributions" and threshold=0), which minimized the number of outliers to around 1% across the topics for all four questions.

**Table 1:** Numbers of documents, generated topics (before and after topic merging), and outliers (before and after reduction) for the four questionnaire items

	Documents	Topics (initial)	Topics (after merge)	Outliers (initial)	Outliers (after reduction)
Q1	748	15	8	207	1
Q2	774	11	8	254	5
Q3	774	16	14	288	7
Q4	771	54	10	230	4

### Participant recruitment and demographics

We note that the online self-care deployment (Sect. 3.1) is publicly available online. During the active recruitment phase for this study, we recruited participants by two means: (1) advertisements posted around our university campus to recruit students, and (2) *Prolific*, an online human subject pool that connects researchers and prospective study participants for high-quality academic studies [78]. By using Prolific, we were able to diversify the participant pool and gain more data to analyse.

Of the total 810 participants we recruited, 538 came from the local campus at the University of Oulu between May 23, 2020, and January 12, 2021—a period that overlapped with national COVID-19 restrictions in Finland—while 272 were recruited via Prolific between August 29 and 31, 2022, after most pandemic-related restrictions had been lifted. Among the University of Oulu participants, we raffled sets of movie tickets, while Prolific participants received US$3.8 as compensation for their participation. All participants recruited through Prolific were required to be fluent in English, current students in a higher education institution, and have completed at least 50 tasks on Prolific. Prolific participants belonged to 24 countries, with the largest proportions from the United States (n = 53), South Africa (n = 45), and Portugal (n = 33). The majority of participants reported English as their language (n = 126), followed by Spanish (n = 37) and Portuguese (n = 33). Full distributions of country of residence and language are provided in “Appendix 3”. Participants’ ages ranged between 18 and 70 years ($$M = 25.24$$ years, $$SD = 6.64$$ years), with 344 males, 449 females, and 17 non-binary or those who preferred not to state their gender. While our sample contained a few older participants, their median age of 23 aligns with the expected age of higher education students. The participant sample had diverse academic backgrounds, representing various fields such as natural sciences, humanities, economics, law, psychology, architecture, and others. Most participants were bachelor-level students (n = 446), followed by master-level (n = 242) and doctoral-level (n = 23) students. The rest (n = 99) did not provide information about their education level. These cases were included in the data, as education level was not a factor in the conducted analyses. Although our sample spans a broad age range and includes bachelor’s, master’s, and doctoral students, we pooled them into a single group because our focus was on the general higher education student population.

## Results

**Fig. 3:**
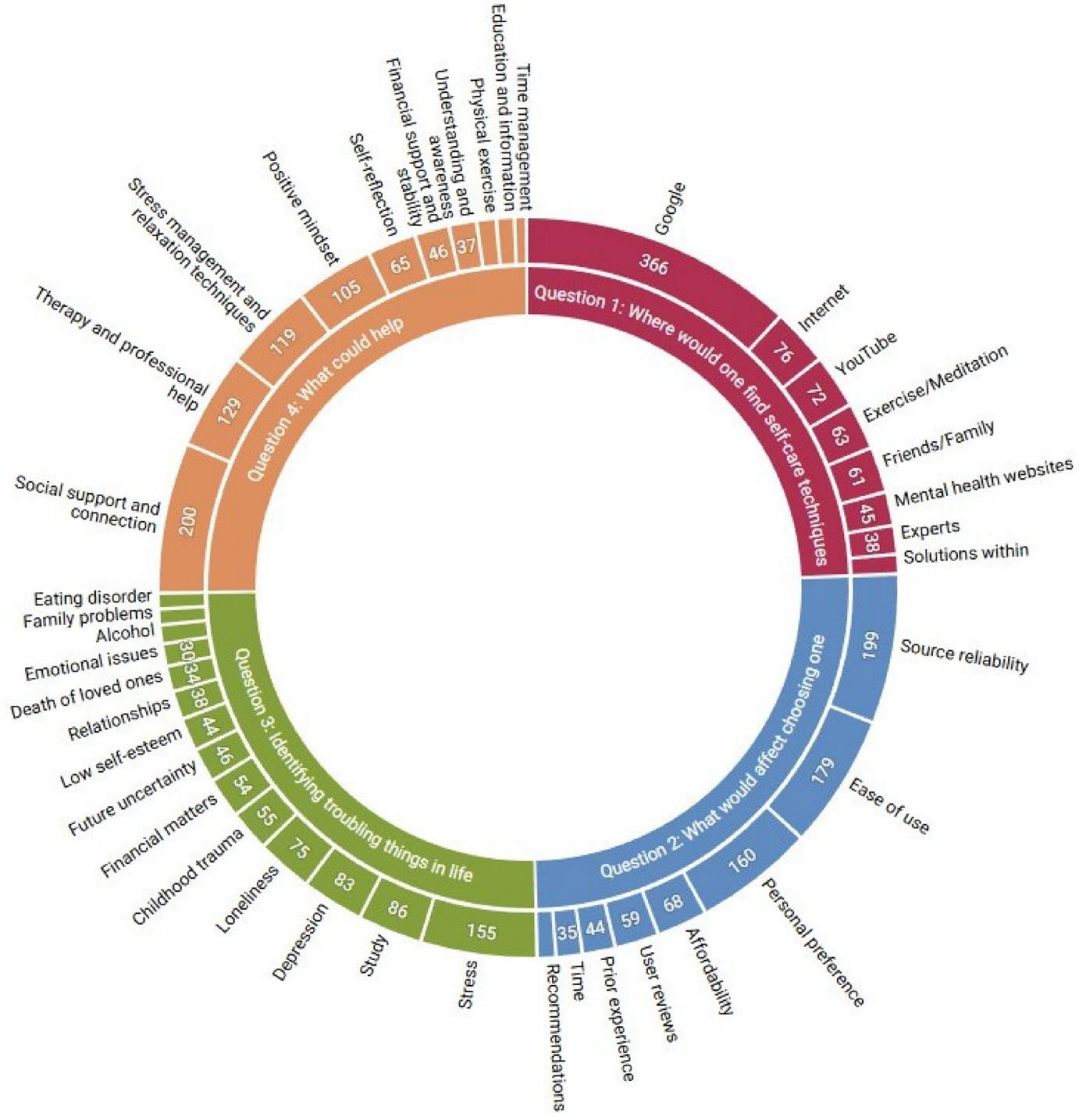
Overview of topics for all four questions. The size and number of each topic slice indicate the count of responses associated with each topic

Following topic generation with BERTopic, we manually inspected the documents and top keywords within each topic to identify duplicates or highly similar topics and applied the BERTopic function merge_topics() to combine them. For Question 4, BERTopic initially generated 54 topics, as we set the min_cluster_size parameter (Sect. 3.4) to a small value of 3. This was necessary, as higher values resulted in too few topics for this specific question. Merging similar topics reduced the number from 54 to 10 (Sect. 4.4). “Appendix 2” provides details of the original topics along with their manually selected groupings and labels. Topic merging and labeling were initially carried out by the first author through manual inspection of the documents in each topic and subsequently reviewed and refined together with two other co-authors. The authors negotiated until consensus was reached, case by case. As topic modeling is inherently exploratory, these outputs should be interpreted as indicative themes rather than definitive categories. Across all questions, the final topic distributions and corresponding document counts—including the effects of outlier reduction and topic merging—are summarized in Table 1, and an overview of topic distributions is shown in Fig. 3.

### Q1: Finding self-care support for mental wellbeing

BERTopic analysis of the first question identified 15 distinct topics related to where students seek help to find self-care techniques. The total number of topics was reduced to eight after merging similar topics. Next, we reviewed the documents within each topic to assign descriptive labels. We applied the same process to the rest of the questions in the subsequent sections. In each topic, a main subject is predominant, yet some unrelated documents are still present. The first topic, ‘Google’, had the highest number of documents with 366 entries, followed by ‘Internet’ (76), and ‘YouTube’ (72). In the following subsections, we provide a breakdown of each topic.

#### Google (n = 366)

The primary theme in the first topic is using Google to search for self-care techniques. Many documents also mention other sources, such as YouTube, Reddit, Instagram, TikTok, Pinterest, and Tumblr, as valuable for self-care ideas. One student noted, “*I usually look online, websites and social media. I follow quite a few people on Instagram and Tumblr who share mental health tips and coping strategies.*” (Female, 24, Master of Education and Learning).

#### Internet (n = 76)

This topic primarily centered around keywords like “*internet*”, “*online search*”, and related terms, with most responses emphasizing searching, making it a more general extension of the first topic. However, some participants provided more detailed responses, such as exploring websites, blogs, forums, and social media platforms.

#### YouTube (n = 72)

Many of the documents in this topic were concise, featuring responses as “*Google or YouTube*” or “*I would search from YouTube*”. Some participants expressed trust in YouTube videos, as indicated by statements like, “*I would try to find a video on YouTube as there are often good videos to all kinds of topics on that platform*” (Male, 30, Bachelor of sports management). However, some expressed scepticism. One participant stated, “*Well certainly I would Google it first. My second guess would be YouTube—there will always be some bad advices but I would be willing to try something out and see how it fits my life.*” (Male, 27, Master of Media Informatics).

#### Exercises/meditation (n = 63)

This topic focuses on the reliance on physical activities like exercise, meditation, and health-related practices such as sleep, nutritious food, vitamins, yoga, and sports. Participants sought meditation guidance from sources like Google, social media, and books. Some explored unconventional sources: “*I’d visit dark web and buy ketamine or mushrooms with psilocybin. AFTER reading actual research about them and how to safely do that. Also Google some meditation practices suitable for me.*” (Gender unknown, 25, Humanities).

#### Friends/family (n = 61)

Participants mentioned turning to family and friends for self-care guidance. One participant stated, “*I would first ask friends and family if they have found any techniques that have been helpful. Depending on the answers I would then do research on those techniques to see which ones would best suit my needs.*” (Male, 34, Master of Business Management). This individual seeks validation through online research after receiving input from loved ones. In contrast, many participants rely on their friends and family without external validation. For instance, one participant stated, “*Talk to friends and family and follow their suggestions.*” (Female, 32, Anonymous study).

#### Mental health websites (n = 45)

Participants reported using search engines, such as Google, to find information and resources on self-care techniques. They specifically sought out websites from mental health organizations, healthcare providers, and other reputable sources offering self-care suggestions and strategies. These websites were regarded as reliable tools for enhancing mental health.

#### Experts (n = 38)

Participants expressed a preference for contacting healthcare professionals, such as counsellors, nurses, psychologists, or doctors. Some participants mentioned using school resources, such as student health centres and counsellors. For instance, a participant (Female, 22, Physics) stated, “*I would go to school doctor and tell my problems. I wish that doctor could help me to go psychologist or somebody else.*”. Trust in the source emerged as a critical factor in seeking self-care support, influencing participants’ decisions to opt for professional help. As one participant (Male, 21, Bachelor of Engineering) stated, “*I would take advice by trusted friends or my doctor only*”.

#### Solutions within (n = 26)

Participants expressed a preference for assuming personal responsibility for their self-care, rather than relying on external assistance. Many emphasized the importance of identifying activities or environments that provide peace and comfort, such as spending time outdoors, engaging in hobbies, or practising mindfulness. They highlighted the role of personal experiences and preferences in selecting self-care practices. Taking breaks, exploring new places, meeting new people, and pursuing hobbies were mentioned as effective strategies.

### Q2: Rationale for selecting self-care techniques to try

The BERTopic analysis of the second question identified fourteen topics related to factors influencing students’ choices of self-care techniques. While there was a certain level of overlap between the topics, each topic primarily represented a dominant subject besides some secondary ones. Each topic’s documents were manually examined and assigned a label that best describes the documents. The most popular topics were ‘Source reliability’ (199), ‘Ease of use’ (179), and ‘Personal preference’ (160). In the next subsections, we present a detailed explanation of each topic.

#### Source reliability (n = 199)

Participants’ emphasized the importance of scientific proof and reliable sources in their decision-making. Trusted sources include official health websites, healthcare professionals, and reputable organizations. “*I find scientific articles to be the best proof of concept there is for any type of thing, be it nutrition, engineering or mental health techniques. You do have to make sure that you qualify in the study group on which the article idea is experimented on. If not, there may be a chance that it won’t work for you.*” (Male, 23, Master of Computer Engineering). Additionally, participants expressed scepticism towards pseudoscience and the biases surrounding self-care recommendations.

#### Ease of use (n = 179)

Participants expressed a preference for self-care techniques that are easy to understand and implement, can be executed quickly and with minimal effort, and do not require expensive equipment or significant lifestyle changes. They also value techniques that can be practiced privately at home, accessed online, or learned from reputable sources. “*Something that I can do easily and quickly, without much effort and time. Something that is affordable and can be done from home. Also techniques that can be used in public could be useful, too.*” (Female, 26, Master of English Philology).

#### Personal preference (n = 160)

Participants identified personal preference as a key factor influencing their choice of self-care techniques. Their input revealed a range of factors associated with personal preference, including feelings, moods, situational considerations, and self-awareness. Some participants selected activities or hobbies they are interested in practising as self-care techniques, such as exercise, meditation, nutrition, and stress avoidance. “*What I’d feel more comfortable and economic*” (Male, 23, Marketing).

#### Affordability (n = 68)

Participants frequently mentioned price and accessibility as factors influencing their choice of self-care techniques. Some indicated a preference for techniques that are entirely free, for example “*Price, if it costs money I will probably not use it*” (Female, 25, Master’s degree—Unknown faculty), “*Free, good instructions, possibly video*” (Female, 29, PhD in Archaeology).

#### User reviews (n = 59)

Here, online user reviews on self-care techniques are a decisive factor in technique selection. One participant (Male, 21, Bachelor of Computer Science) said “*Number of up votes on a post with a book recommendation. Frequency of the recommendations of a certain book. If it shows up in multiple searches then it has a higher chance of being picked by me.*”. Factors like comments, feedback, and recommendations contribute to perceived source authenticity. The opinions and experiences of professionals, patients, and online users are regarded as influential.

#### Prior experience (n = 44)

Participants value stories and experiences from friends, acquaintances, and others who have successfully tried the technique. Recommendations and opinions from trusted individuals are also influential. They prefer authentic, personal narratives over professional advice, seeking individuals with similar challenges who have found success with the technique. This topic intersects with both ‘User reviews’ and ‘Recommendations from others’ topics. One participant says, “*How exactly it helped others—i.e. how they were feeling before, during and afterwards. How they think it helped them and how they (can) apply it in their day to day.*” (Female, 38, PhD in Medicine).

#### Time (n = 35)

Participants prioritize techniques that are not overly time-consuming, complicated, or physically demanding, favoring options that can be performed at home. This topic correlates with the ‘Ease of use’ topic, as evident from participants’ responses that often highlight the combination of time and simplicity, such as one participant stating, “*The time it takes up to do and the simplicity of the technique.*” (Male, 20, Politics). Another participant expresses, “*How tedious or time consuming it might seem, I’m not a very disciplined person, I don’t think I can keep up with something too complicated or that takes me too far away from what I normally do.*” (Male, 32, Master of Civil Engineering).

#### Recommendations (n = 25)

Recommendations from friends, family, peers, and professionals significantly influence the choice of self-care techniques. “*If my friends have tried it and felt it to be effective or a recommendation from a trustworthy person to me would both make me more interested and willing to try*” (Female, 22, Medicine). Participants also consider the effectiveness and success stories shared by others who have tried the technique very important.

### Q3: Issues degrading mental wellbeing in higher education

The BERTopic analysis of the data addressing issues affecting students’ mental health identified 15 distinct topics, with no clear rationale for merging any of them. The most prevalent topics are ‘Stress’ (155), ‘Study’ (86), ‘Depression’ (83), and ‘Loneliness’ (75). While several topics are related, e.g. ‘Loneliness’ and ‘Depression’, we examine each topic separately to uncover more comprehensive insights.

#### Stress (n = 155)

Many participants identified stress as a key concern, accompanied by factors such as lack of motivation, fatigue, and difficulty managing daily tasks. Participants attributed their stress to multiple factors, including seasonal influences like winter, limited relaxation time, a perception of underachievement, and poor sleep quality. One participant consolidated multiple stress factors by stating “*Mostly stress and anxiety regarding, for example, the climate crisis, school and studies, work, relationships with friends and family, health, money, the political state of the world, spare time activities and so on*” (19, Female, Bachelor of Social Sciences).

#### Study (n = 86)

Many entries on this topic highlight the pressures of academic performance, deadline adherence, and workload management. One participant elucidates the challenges associated with study and work:, “*Stress related to study and work has been a huge factor. Students in this country are placed under immense strain with the pressure to perform as best you can and meet every deadline and expectation. This is massively intensified by the financial aspect of our decision to enter student life due to insurmountable fees and living costs. It feels like every decision you take is automatically subject to intense financial scrutiny, and any second not spent ‘making the most of your time’ (in whichever way that may be) is a waste of money and time.*” (Male, 24, Bachelor of Education).

#### Depression (n = 83)

Many participants reported experiencing severe depressive episodes or being diagnosed with clinical depression, characterized by sadness, low mood, lack of motivation, and difficulties in task completion or enjoyment. One participant attributed his depression to financial factors, “*Depression which is caused by rising inflation and stagnant income gives me mental issues, such as stress, inability to focus and headaches.*” (Male, 24, Bachelor of Information Systems). Other participants associated their depression with specific disorders like bipolar disorder, panic disorder, and trauma, while others attributed it to burnout, stress, and unpleasant life situations.

#### Loneliness (n = 75)

Several entries on this topic reveal feelings of loneliness, social isolation, and difficulty in forming or maintaining relationships. Loneliness arises in various contexts, such as during the COVID-19 pandemic, post-breakup scenarios, social anxiety, and difficulties faced by introverted individuals. One participant shares, “*Being alone and the feeling to be left alone. I think that is the main concern and all the other concerns are linking to that. I have had experience of being outside of groups, and actually the situation has actualized in this extraordinary time of distant studying. It makes me think if I am somehow mean or just boring person to hang around with. Luckily there are better days when I see my oldest friends and notice they are still here for me and I for them.*” (Male, 21, Bachelor of Law). His words concluded on a positive note, highlighting the importance of supportive friendships in alleviating loneliness and fostering a sense of belonging.

#### Childhood trauma (n = 55)

Numerous participants openly shared enduring bullying, particularly during their school years. Additionally, many disclosed experiences of childhood sexual abuse, leading to profound frustration and anger towards their abusers. One participant articulated their struggles, stating, “*I suffer how mean and cruel people can be. I hate malicious people. Being bullied at school, being sexually abused, bad relationships (both friends and dating), money, seasonal depression, being very demanding towards myself... and fucking covid-19 and online school. I hate it, I hate not knowing when things get better, I hate not seeing my school mates.*” (Female, 27, Bachelor’s in Social Services). Other participants reported feelings of diminished self-worth and difficulty fitting in or forming friendships.

#### Financial matters (n = 54)

Numerous participants reported financial troubles, including insufficient funds, job insecurity, and concerns about providing for their families. A key aspect of these issues was the difficulty in securing employment, as illustrated by statements like, “*Having the ability to find a job*” (Male, 32, Master of Education) and “*Financial status, search for internship and university.*” (Male, 29, Bachelor of Pharmacy). One participant expressed frustration regarding the disparity between excessive workload and inadequate compensation, “*Too much work and loss of money*” (Female, 38, Master of Health Science).

#### Future uncertainty (n = 46)

Many participants expressed concerns about their future, including career prospects, relationships, personal choices, and an overall uncertainty about what lies ahead. Some fears stem from inadequate planning or a lack of clarity regarding the future. For instance, one participant stated, “*I am a man who often worries. Most often, however, I’m afraid of my future. I think I haven’t found my destiny yet and I don’t know what I want to do in the future.*” (Male, 19, Information Technology). Others expressed negative expectations, such as “*How I would deal with the future. What if I would be alone my whole life? What if I would be unhappy and unsatisfied my whole life? Which path should I choose (by that I mean like a career).*” (Male, 22, Bachelor of Economic Sociology). Additional concerns include a lack of direction, feeling overwhelmed by adulthood, and fears of unrealized potential.

#### Low self-esteem (n = 44)

Several participants referenced low self-esteem, often in conjunction with depression, anxiety, and self-image issues. One participant elaborated on her experience, stating, “*Feeling useless and constantly comparing myself to others and subsequently determining my own self worth based on it. The weight of unfairness and inequality in society, looking around and not being able to understand why some are treated so differently from others purely based on external personal factors that do not affect others.*” (Female, 18, Bachelor of Psychology).

#### Relationships (n = 38)

This topic encompasses multiple dimensions of relationships, including personal, familial, romantic, and toxic relationships. A participant expressed the importance of having healthy relationships, stating, “*Having the right people around you who you could talk to.*” (Male, 21, Bachelor of Engineering). In contrast, another participant highlighted negative experiences in relationships, specifically mentioning “*Toxic relationships and bad influences among friends.*” (Male, 20, Bachelor of Physiotherapy).

#### Death of loved ones (n = 34)

Several participants reported the loss of family members, friends, and loved ones due to accidents, suicide, or illness. While some briefly mentioned these losses, others provided detailed accounts of the deceased and the emotional toll. One participant shared his experience of multiple successive losses, “*The death of my grandfather remains one thing that I do not believe I will ever be able to recover from. This also happened in the same year I got into a car accident, just 6 months later. And 6 months later after the passing of my grandfather, I lost the closest Professor at University, one whom I looked up to. It took me a very long time to accept this, but with the help of my best friend, I was able to come to terms with it. A year later, my best friend also passed on, due to a car accident and before I could even process this loss, I have now lost another friend this week. What pains me, even more, is that I was unable to attend the funerals of both my friends because I did not have money for transport.*” (Male, 27, Bachelor of Humanities).

#### Emotional issues (n = 30)

This topic reveals several emotional challenges, including existential crises and questioning life’s meaning, managing strained relationships and past traumas, and confronting negative self-perceptions often exacerbated by social comparisons. Many participants also reported struggling with external judgments and prejudices, with stigma surrounding men’s mental health remaining a notable concern. Issues with concentration, uncertainty about the future, and feelings of being unheard or dismissed are also prevalent. Moreover, the COVID-19 pandemic has significantly impacted mental health and motivation for many individuals.

#### Alcohol (n = 25)

Several participants reported alcoholism in the family, excessive alcohol consumption, and financial issues linked to alcohol abuse. Additionally, some mentioned substance abuse and addiction, including gambling, cigarette, and alcohol. One participant detailed his traumatic life experiences, stating, “*I’ve experienced a variety of traumatic experiences in life. Growing up there were people in my immediate family with drug and alcohol issues, violence, divorce, jailprison, and lots of relocating all over the country. General chaos and instability.*” (Male, 44, Bachelor of Business).

#### Family problems (n = 21)

This topic reveals a significant influence of family dynamics on mental health. Participants frequently cite family-related matters, such as health problems, conflicts, expectations, and financial situations, as contributing factors to their mental health issues. For instance, one participant described feeling burdened by their family’s expectations for academic success, stating, “*Education and family has had the most impact on my mental health over the years because I’ve had high expectations put onto me since I was a kid. I had a fear of failure since I was the oldest sibling as well as living in a low income household.*” (Male, 19, Bachelor of Computer Science).

#### Eating disorders (n = 21)

Several participants mentioned struggles with eating disorders, including bulimia, anorexia nervosa, binge eating disorder, and fasting disorder. A participant detailed her challenges, stating, “*Trouble sleeping for multiple years. Eating disorder and body image issues. Loss of family member and being left without any mental support at the time.*” (Female, 24, Master of Science). Her response further emphasized the ongoing lack of adequate mental health support.

### Q4: Fostering mental wellbeing: self-care and beyond

The fourth question explored the techniques individuals find helpful for their mental wellbeing. The BERTopic analysis identified 54 topics, a higher-than-expected number due to the small cluster size, as explained in Sect. 3.4. After carefully reviewing the top keywords associated with each topic, these were consolidated into 10 distinct topics. The most prominent topics were ‘Social support and connection’ (200), ‘Therapy and professional help’ (129), and ‘Stress management and relaxation techniques’ (119). A detailed analysis of each topic is provided in the following subsections.

#### Social support and connection (n = 200)

Participants emphasized the importance of friends, family, and a reliable support system. They mentioned the value of open dialogue, sharing experiences, and seeking advice from trusted individuals. Additionally, engaging in social activities, meeting new people, being part of support networks, and opportunities for socializing were seen as helpful ways to combat loneliness and enhance mental wellbeing. “*I believe for any problem someone may face, support from friends and family is of the utmost importance.*” (Male, 25, Master of Computer Engineering).

#### Therapy and professional help (129)

Many participants mentioned the importance of consulting a psychologist, therapist, or counselor for professional mental health support. Additionally, some noted the effectiveness of medication. Moreover, some participants proposed enhancing access to mental health services in educational institutions, offering guidance on time management and workload planning, and fostering a supportive and empathetic environment for students.

#### Stress management and relaxation techniques (n = 119)

Participants highlighted various stress management techniques, including meditation, yoga, breathing exercises, mindfulness, and engaging in hobbies or activities that promote relaxation and stress relief. One participant noted, “*I feel like meditation could help a lot, as that would help me free my mind from anything that could be troubling.*” (Male, 22, Bachelor of Business Management). Another shared, “*Guided meditation, mindfulness, going for a walk, running, personal yoga teacher, breathing techniques*” (Female, 23, Bachelor of Swedish and Literature).

#### Positive mindset (n = 105)

Participants highlighted the significance of developing self-confidence, reframing negative thoughts, practicing self-love, and focusing on the present moment. They stressed the value of accepting imperfections, being kinder to oneself, practicing self-compassion, and letting go of self-judgment. As one participant noted, “*I feel like this kind of issues are solved by turning to ourselves and really accepting and be mentally available to better yourself. For example, by thinking deeply about what it meant, what was only in my head and trying to create new habits of though, different ways to look at them.*” (Female, 20, Bachelor of Psychology). Additionally, several participants mentioned the need to accept help from others and be open to support.

#### Self-reflection (n = 65)

Participants highlighted the importance of self-reflection, self-acceptance, and self-awareness. They mentioned practices like journaling, practicing gratitude, setting boundaries, and focusing on personal growth and wellbeing. One participant expressed, “*I’d like to learn more about myself and what do I expect from friendships, and how to establish boundaries.*” (Male, 23, Bachelor of Pharmaceutical Science). Moreover, some participants expressed a desire for self-analysis, self-discipline, and gaining a better understanding of their emotions and behaviors.

#### Financial support and stability (n = 46)

Some participants highlighted the importance of securing a stable employment and addressing financial concerns to alleviate stress and enhance mental health. One participant shared, “*I believe if someone were to help me the person would offer me a job or a financial solution to my problem maybe a modern way to make money like online money-making.*” (Male, 21, Bachelor of Tourism Management). Another participant sarcastically noted that financial abundance could solve many problems, remarking, “*If someone would give me a high paying job or 50 million USD most if not all my troubles would disappear.*” (Male, 23, Bachelor of Engineering). However, several participants also expressed the difficulties they encounter in securing employment.

#### Understanding and awareness (n = 37)

Many participants expressed a desire for greater understanding of the challenges they face as higher education students, such as social anxiety, academic stress, and financial pressures. One participant shared, “*opportunities to meet like-minded people, other people having a better understanding and awareness of social anxiety and how it can affect a person (not immediately thinking that I’m stuck up/disinterested/rude/boring/always quiet)*” (Female, 25, Masters of English).

#### Physical exercise (n = 26)

Physical exercise, maintaining a healthy lifestyle, and caring for one’s body through proper diet and sleep were mentioned as important factors for mental wellbeing. “*Sport has always helped me in such situations. Right physical effort more accurately. I reset my head and immediately felt better.* (Male, 21, Bachelor of Administration). Additionally, participants emphasized the importance of establishing regular, healthy sleep patterns.

#### Education and information (n = 24)

Participants highlighted the importance of access to mental health education, emotional skills training, and knowledge of various coping mechanisms through therapy, self-care literature, or online resources. Some notes the advantages of understanding their thoughts and emotions while gaining insights into self-improvement techniques. One participant articulated this need, “*Easy access to (mental) health services, more mental health and general life education throughout all stages of compulsory and optional schooling (learning to listen to your needs, identify your feelings, self care, establishing healthy habits, value driven decision making, dealing with hardship, accepting emotion, mindfulness...). I think everyone should attend therapy sessions at some point in their lives.*” (Female, 25, English Philology).

#### Time management (n = 16)

Some participants expressed the need for improved time management, organization skills, and the development of effective schedules to alleviate stress and manage workloads. They also emphasized the importance of dedicating time to self-care and establishing boundaries to foster a healthier work-life balance. One participant suggested, “*Some guided time management, relaxing tips and techniques. Also some counselling and guiding in life decisions and relationship troubles. Tips for studying and getting a job.*” (Female, 26, Masters of English Philology).

### Gender differences across topics

**Fig. 4:**
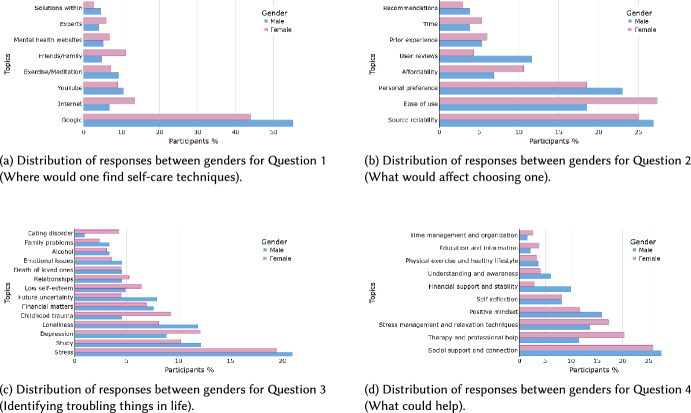
Distribution of participant responses by gender across topics for each of the four questions

Gender differences are evident in the disclosure of mental health issues and the selection of self-care techniques [21]. In our sample, after removing invalid and duplicate entries, the distribution of participants by gender is as follows: Male = 335, Female = 421, Non-Binary/Unknown = 18. Figure 4 illustrates the percentage distribution of participants’ genders across the topics of the four questions. We excluded the Non-Binary/Unknown group from analysis due to its smaller sample size. Consequently, the gender-based findings pertain only to male and female participants, and we acknowledge this as a limitation, cautioning against over-generalizing results to non-binary or other gender identities. We conducted chi-square tests of independence for each question to examine gender differences. To account for multiple testing, *p* values were adjusted using the Benjamini–Hochberg false discovery rate (FDR) procedure. Effect sizes were quantified using Cramér’s V, with values indicating small-to-moderate associations. The results, summarized in Table 2, show that all four questions exhibited statistically significant differences between male and female participants after FDR correction.

**Table 2:** Gender dependency results for the four questionnaire items using chi-square test of independence, including effect sizes, 95% confidence intervals, and FDR-corrected *p* values

	$\chi ^2$	df	N	*p* (raw)	*q* (FDR)	Cramér’s V	95% CI
Q1 (Where would one find self-care techniques)	25.35	7	729	0.0007	0.0009	0.186	[0.075, 0.239]
Q2 (What would affect choosing one)	25.75	7	751	0.0006	0.0009	0.185	[0.085, 0.278]
Q3 (Identifying troubling things in life)	24.29	13	750	0.0286	0.0286	0.180	[0.097, 0.250]
Q4 (What self-care techniques could help)	32.91	9	749	0.0001	0.0006	0.210	[0.098, 0.313]

Observing the plots, it appears that females are more likely to seek help through personal networks (such as friends and family) and mental health websites. They prioritize techniques that are affordable and easy to use, experience higher levels of emotional challenges such as depression, childhood trauma, and eating disorders, and place greater value on professional therapy and social support. In contrast, males tend to be more specific about the search platforms they use to obtain information, such as Google and YouTube. They are influenced by user reviews and personal preference when choosing self-care techniques. Moreover, males experience more stress, future uncertainty, and loneliness. They emphasize financial stability and a better understanding of their issues as key supports for managing their wellbeing.

### PHQ-8 scores across topics

**Fig. 5:**
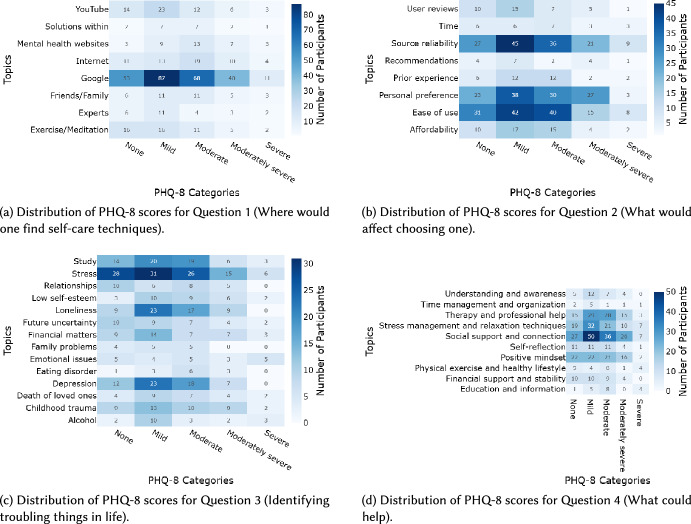
Distribution of participant PHQ-8 scores across topics for each of the four questions

The heatmaps in Fig. 5 illustrate response concentrations across different topics in relation to participants’ PHQ-8 scores. In our sample, after removing outlier topics and including only entries from participants who completed the optional PHQ-8 questionnaire, the numbers of participant entries used in the heatmaps were as follows: Q1 = 540, Q2 = 558, Q3 = 555, and Q4 = 555. PHQ-8 scores were grouped into categories using the commonly accepted depression ranges: 0–4 (none/minimal), 5–9 (mild), 10–14 (moderate), 15–19 (moderately severe), and 20–24 (severe) [57]. Although the chi-square test of independence results (Table 3) indicated no significant association between PHQ-8 scores and topics across all questions, the heatmaps reveal observable patterns in the data. For instance, participants reporting moderate to moderately severe stress symptoms showed a preference for social and professional support as their preferred help source (Fig. 5a). Similarly, for the question "What is troubling you?", participants with higher PHQ-8 scores tended to report challenges related to ‘stress’, ‘loneliness’, ’childhood trauma’, and ‘study’ more frequently (Fig. 5c). These descriptive trends should be interpreted cautiously and do not imply statistically significant associations.

**Table 3:** PHQ-8 scores versus topics dependency results using chi-square test of independence, including effect sizes, 95% confidence intervals, and FDR-corrected *p* values

	$\chi ^2$	df	N	*p* (raw)	*q* (FDR)	Cramér’s V	95% CI
Q1 (Where would one find self-care techniques)	22.69	28	540	0.7483	0.7483	0.102	[0.118, 0.188]
Q2 (What would affect choosing one)	22.90	28	558	0.7380	0.7483	0.101	[0.116, 0.186]
Q3 (Identifying troubling things in life)	58.67	52	555	0.2443	0.4886	0.163	[0.184, 0.264]
Q4 (What self-care techniques could help)	49.79	36	555	0.0629	0.2516	0.150	[0.153, 0.242]

### Flow of participant responses across topics of the four questions

**Fig. 6:**
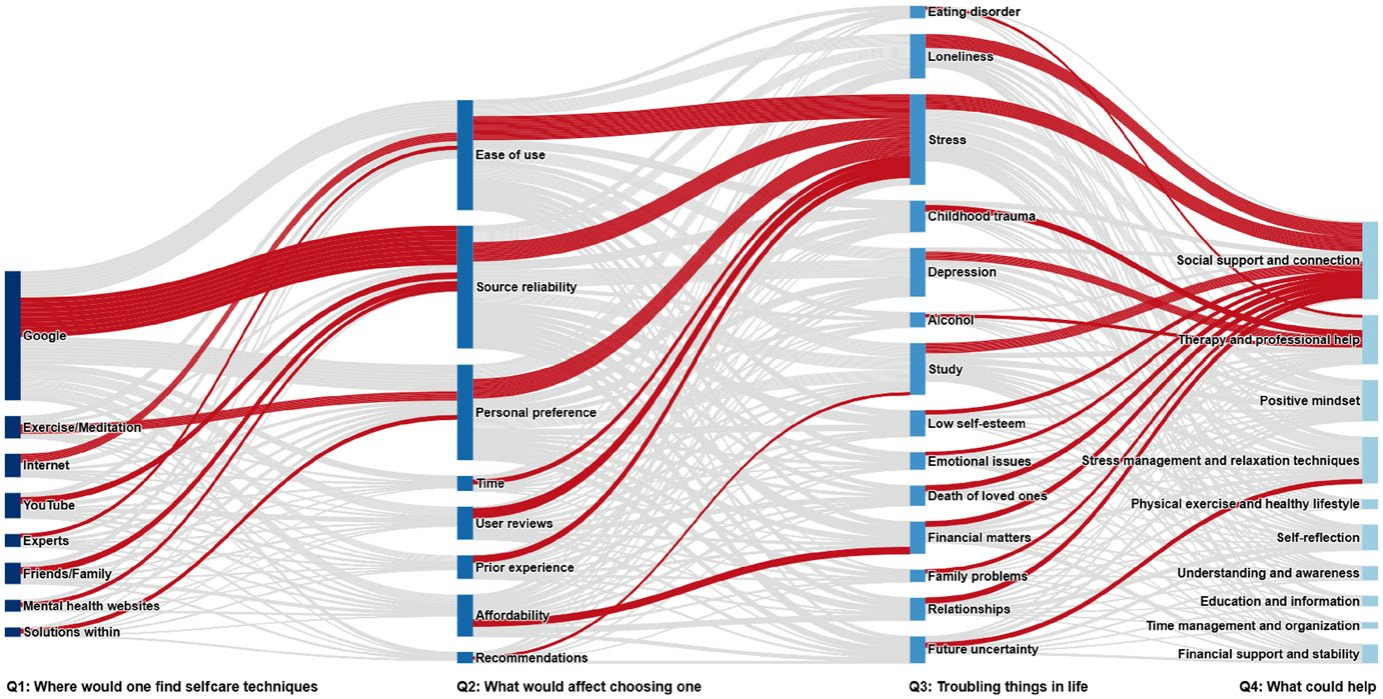
Flow of participant responses across topics. Red connections indicate the dominant flow(s) per source topic based on absolute participant counts. See “Appendix 4” for the top three flows per source topic

To gain an understanding of the relationships between participants’ responses across the four questions, a Sankey diagram (Fig. 6) was created to visualize the flow of responses from the topics of each question to the next. For each source topic, the dominant flow(s) highlighted in red were determined based on the absolute number of participants transitioning to the next topics. In the case of multiple outgoing flows having the same maximum count, all would be highlighted as dominant (tie resolution); in our dataset, no such ties occurred. Supplementary “Appendix 4” provides the top three flows per source topic, including counts and percentages.

#### From question 1 to 2

Most online resources, such as ‘Google’ and ‘YouTube’, were strongly linked to the topic of ‘Source reliability’. This indicates that individuals place a high value on validation and credibility when seeking information online. In contrast, self-guided sources like ‘Solutions within’ and ‘Exercise/Meditation’ were connected to ‘Personal preference’, emphasizing the importance of autonomy and individual relevance in their decision-making.

#### From question 2 to 3

‘Stress’ emerged as the dominant connection for most selection criteria, highlighting its role as a primary concern affecting students. A practical link between ‘Affordability’ and ‘Financial matters’ emphasises the importance of affordable or free resources, reflecting the financial constraints many students face.

#### From question 3 to 4

the majority of topics, including severe ones, such as ‘Stress’, ‘Loneliness’, and ‘Study’, were connected to ‘Social support and connection’. This reflects a widespread recognition of the importance of social support in promoting mental wellbeing and guiding self-care. However, ‘Therapy and professional help’ emerged as a key solution to more severe issues, such as ‘Childhood trauma’, ‘Depression’, and ‘Alcohol’, highlighting the crucial role of professionals in addressing persistent and complex mental health challenges.

## Discussion

Healthcare is globally in a resource crisis. And this shortage extends to mental health resources in higher education. More and more attention is being paid to not just reacting to already manifested issues but proactively turning to design for maintaining mental wellbeing [102]. For example, specifically with the university student population, Tag et al. [98] found that in situations of mental distress and isolation, students increasingly turn to digital tools when traditional offline strategies are less accessible. Technological or scientific therapy-based interventions have been proposed as promising strategies to address access disparities and provide scalable support [54]. To this end, a variety of digital mental wellbeing solutions have been explored recently (see, e.g.  [73, 90, 113]).

In our work, we focus on two different questions to understand more about mental wellbeing self-care in higher education. First, we aimed to explore the self-care strategies that the community looks for, or would go looking for, to enhance wellbeing. Second, we investigated the current challenges students face, as well as the approaches they find helpful, or feel optimistic about, in addressing these challenges. Through understanding these fundamentals, we can then discuss how recent technological advancements can contribute to facilitate self-care for higher education students.

### Understanding mental wellbeing self-care in higher education

Much of mental wellbeing is subjective, personal and tied to the individual’s situation, but simultaneously shaped by the person’s current circumstances [41].

To answer the first research question (RQ1), we analysed where and based on what the participants find new self-care techniques in their unique life situations. In terms of finding new self-care techniques, it is evident that higher education students are an Internet-savvy demographic, with online search in all its various forms (Google, YouTube, Reddit, Instagram, and TikTok) being the most popular approach. In the end, even the topic classified as exercises and meditation mentioned mostly finding these exercises online. Other sources included healthcare professionals, such as school doctors, but they were nowhere near as frequently mentioned as online search, much in line with [31, 40, 86]. Regarding self-care in higher education, online search in many forms and modern social networking sites—not just the one dominant search engine—is key.

For virtually any online search, a plethora of options is available to choose from. Making sense of those options and deciding on something is just as important as finding them in the first place. For our demographic, some important factors to consider are money and time, as higher education students often do not have too much of either. Our participants are looking for cheap and fast ways to do this. Other, perhaps less surprising, factors were favourable reviews or experiences by other people and source reliability, referring to some scientific or other formal validation of the information.

Overall, our data is largely in line with findings among more general populations and thus helps build validity [5, 83] in terms of how they would go about choosing new self-care techniques while also highlighting the issues in higher education.

To answer the second research question (RQ2), we analysed higher education students’ current concerns and ideas on potential solutions. Here, the data tells about a concerning situation among the population. First, loneliness and the pandemic were major issues among students. In addition to the earlier point about affordable solutions being favoured, financial matters were mentioned both explicitly and in conjunction with student life in general. In line with these considerations, social support systems and connections were highlighted as something that could help with feelings of loneliness.

Affordability is a recurring theme. The poor financial situation is also clear through the suggestions about simple mechanisms like going for a walk or breathing techniques or simply self-reflection, which were seen as good ways to remedy some of the distress. Almost all of the key themes, aside from “financial support and stability”, deal with relatively affordable methods.

The questionnaire analysis provided a more in-depth look into both research questions (RQ1 with Sects. 4.1 and 4.2, and RQ2 with Sects. 4.3 and 4.4). Yet the issues discussed above are what we can isolate as being particularly topical for higher education, as they were mentioned across multiple different themes. These issues translate into logical design implications for digital self-care systems in higher education.

### Implications for self-care system design in higher education

We discuss design considerations based on the data analysis for future self-care systems intended to support higher education students. To make these implications decision-relevant, we outline what the findings suggest, who the stakeholders are, and which decisions could plausibly change in practice. The primary stakeholders include (1) students as end-users of self-care tools, (2) higher-education institutions (e.g., campus wellbeing units, student affairs offices, and counseling services), (3) those designing digital mental health platforms. The guidelines are based on studying the topics under each of the four questions while focusing on their specificity and real-world applicability, critical for deriving implications from study findings [107]. Where appropriate, we support the considerations with relevant literature from related fields.

#### Ensure credibility of online self-care platforms

Given students’ proclivity for seeking self-care advice online as highlighted in Sect. 4.1, it is imperative that self-care systems are not merely accessible but carefully consider and communicate credibility aspects and/or scientific or other type of reliability of the information provided. This is also supported by answers to Q4 (see Sect. 4.4), in particular with references to therapy and professional help, highlighting the demand for credible and professional help. For product teams, this finding implies a design decision to build explicit credibility cues (e.g., validation badges, transparent sourcing, expert endorsements) that help students quickly assess trustworthiness. For higher-education institutions, it suggests that endorsing or curating a set of reliable digital tools could reduce the cognitive burden on students navigating a crowded and uneven online landscape.

By definition, self-care is not, however, only about acting on advice from authorities. Researchers have built collaborative self-care systems through different crowdsourced approaches. For instance, people affected by a given medical condition can contribute a list of self-care techniques that are then cross-evaluated by clinical professionals [47]. This helps verifying and rating the information, even if the source is by nature not necessarily credible. This also aligns with another design consideration, the need for social and professional influence.

#### Incorporate social and professional influence

Given the powerful influence of social and professional networks on student decisions (Sect. 4.1), we encourage researchers to explore incorporating various options for communication with peers [76], family members, and professionals. Here also responses to Q4 (Sect. 4.4), particularly people mentioning ways to connect to other people as a coping mechanism, speak for the importance of social features.

Collaborative systems that take into account the views of different types of caretakers, and not only clinical authorities, have been shown as useful, e.g. for self-care of Parkinson’s Disease [58]. Collaborative platforms that enable sharing experiences and recommendations can provide a sense of community, and access to expert help can ensure reliable guidance. *MINDNOTES* [16] is an example mobile app that facilitates connections between users and mental health professionals. And while these kind of professional connections, even if more casual, are of course laborious and expensive to maintain, recent HCI work has suggested they have a role in promoting empathy and closeness between different actors, e.g., between patients and their carers Martikainen et al. [66]. Thus, it remains an interesting research challenge to look into models where aspects of professional influence can be integrated into self-care solutions, e.g. in the name of preventive care, thus making it potentially economically viable. Economical feasibility, of course, is also a concern from the user perspective, evident in our data. For platform designers, this highlights a decision to integrate lightweight peer-to-peer features (e.g., anonymous support forums, buddy systems) and options for verified professional input, even if only in a limited form. For universities, it points to the need to create bridges between institutional support (counseling services, advisors) and informal peer networks, ensuring students can access both without stigma or prohibitive cost.

#### Emphasise holistic and affordable solutions

To accommodate the practical constraints of students presented in Sects. 4.2 and 4.3, the design of self-care systems should consider providing cost-effective and easy-to-implement solutions. These needs are further supported in Sect. 4.4, where students emphasized methods, such as meditation, self-reflection, time management and many more that are all essentially free.

A specific challenge here will be to tackle the breadth of needs cost-efficiently: future self-care systems should consider a wide array of student problems, ranging from emotional and psychological issues to academic and financial stress. Integrating free or low-cost methods for specific problems, such as stress management or financial planning, would cater to the diverse needs of students. The problem here, however, is not just availability or the lack thereof. There exists a plethora of free methods that have been scientifically proven to affect mental wellbeing, including mindfulness and reminiscence-based approaches [106]. Perhaps developing more efficient programs to persuade higher education community members to try and adhere to affordable techniques is an equal challenge to building a catalogue of things to try. For policymakers and funders, this evidence suggests prioritizing the scaling of low-cost, evidence-based methods that can be deployed widely across campuses. For university wellbeing units, it highlights a decision point about how to package and promote affordable everyday practices (e.g., guided mindfulness, structured peer reflection) so that students are more likely to adopt and sustain them. For product teams, it could mean a market opportunity for accessible solutions that do not depend on high subscription costs or intensive human labor.

Recent scholarship increasingly frames self-care in higher education as a multilevel construct shaped not only by individual strategies but also by institutional and sociocultural contexts [109]. Our findings reinforce this perspective by showing how students’ choices are constrained by affordability and credibility concerns, and by their reliance on digital platforms over institutional support. Positioning our results against this updated view clarifies the added value: we provide empirical evidence of how these contextual factors manifest in everyday decision-making. Professional ethics further amplify these implications. Higher-education institutions have responsibilities to avoid shifting the burden of wellbeing entirely onto students. Product teams developing digital mental health tools have an ethical duty to communicate credibility transparently and design for equitable access.

### What can technology designers contribute to mental wellbeing in higher education

While computational tools expedite the identification of trends and topics, and people by providing ubiquitous access through, e.g. mobile tools [64], human experts offer essential validation, natural support, critical context, and interpretation of findings. Thus, we believe hybrid systems that leverage the best parts of humans and computers will be useful for mental wellbeing in this context too. Recently, e.g. Large Language Models (LLMs) have been successfully leveraged to help self-guided mental health interventions through the principles of Cognitive Behavioural Therapy [88], among other approaches. More broadly, many kinds of generative AI [35] (GenAI) solutions are expected to enhance self-care practices by providing personalized content tailored to one’s specific needs and preferences. GenAI models could analyse individual user data, including browsing history, social media interactions, and preferences, to recommend relevant self-care techniques and resources. Similarly, they can be trained to provide reliable and safe methods, and that is a challenge on its own that needs to be addressed in the future. However, building any such solutions will need to focus on the credibility of self-care solutions, a key implication in our work. As evident in our data too, students often turn to social media for guidance, and self-care systems should prioritise integrating features that communicate reliability, supported by scientific evidence or vetted by professionals, to counter misinformation and enhance trustworthiness.

Smith et al. highlights how the voices of all stakeholders are critical to listen to and incorporate at all stages of developing algorithmic solutions [92]. This is regrettably not always the case. For instance, research conducted with existing datasets or with social media often omits these considerations (see, e.g., [101]). Incorporating the voice of the eventual end-users is imperative also from an ethics perspective [14]. Deployments like ours, online and constantly collecting new data, can provide some of these voices. Further, this happens in near real-time as the data can be computationally analysed and consensually donated by the actual end users who benefit from the built systems. In our case, we are able to make sense of both the pressing issues that demand attention and the exact ways of where and how people look for help. This kind of information is useful to authorities too, and we argue (in line with the second implication) that collaborative self-care systems that draw on peer support and professional input, such as community-based platforms and professional advice channels, could further enhance online digital wellbeing solutions.

Lastly, there is the growing concern around deepfakes and AI-generated content more broadly, especially when combined with algorithmic recommendations. This leads us to the increasing importance of AI literacy among internet and social media users. The self-care methods people encounter on TikTok are not, for the most part, recommended based on their scientific or even community-based evidence but on engagement metrics and by black box recommendation algorithms. And to begin with, it should be clear that, e.g., looking for self-help from TikTok or Instagram can be seen as an issue itself. To this end, technology researchers should continue to make progress in, e.g., developing scalable ways to understand the spread of misinformation on such platforms [22, 37, 111]. Currently, there is also an emphasis on strategies to teach critical reading and foster media literacy and digital citizenship skills, ensuring individuals are better equipped to evaluate the credibility of sources such as those encountered in our study [24]. By integrating insights from cognitive science, ethics, and design, technology researchers can contribute strategies that can educate about information credibility. This presents another line of work on educational approaches to help people be critical about recommendations and understand the potential biases that AI-based recommendation models in social media come loaded with.

### Pandemic-specific patterns in mental wellbeing

While our data collection overlapped with COVID-19 restrictions, analysis of the topics revealed both pandemic-specific drivers and patterns likely to persist in post-pandemic higher education contexts.

The effects of the pandemic have been documented widely (see e.g. [91] for a comprehensive review concerning the general population), and in our study, we see ripples of these as well. Stress, as the clear top mental wellbeing challenge, was likely exacerbated by the unprecedented simultaneous convergence of general health anxiety, future uncertainty, and social disruption that all were present during the pandemic era. We also know how *loneliness* is common among all, but its prominence in our data most likely reflects pandemic-imposed social distancing, campus closures, and reduced face-to-face interactions that amplified these social challenges. Additionally, students’ heavy reliance on *online resources* like Google and YouTube for self-care guidance may have been intensified by the increased time spent at home and the closure of on-campus services during the pandemic. These are well in line with related work examining the effects of the pandemic era, particularly among higher educations students [94, 97].

The other identified patterns likely extend and generalise well to contemporary higher education, after the pandemic era. For example, the students’ concerns about financial matters and future uncertainty (e.g. about career choices) reflect issues common to these populations worldwide and across different periods in history. Students have never been the richest demographic. Other elements of *study-related stress*, such as deadlines, performance anxiety, and feeling overloaded with work, represent steady features of higher education rather than issues specific to the pandemic. Likewise, students’ preference for *online mental health resources* likely reflects generational and convenience factors rather than something attributable to the pandemic era alone. These exemplify the common and prevalent issues in higher education more generally and are naturally observed in other large-scale studies as well, such as  [48].

Thus, many of the themes we discuss were not directly caused by the pandemic but were influenced and intensified by the crisis conditions. Rather than viewing this strictly as a limitation, our data provides valuable documentation of mental wellbeing in the student population during an unprecedented crisis. This specificity offers unique scientific value: We have captured authentic voices from students who had to face rare circumstances during their studies. Such data alone is precious as a document of history and for providing future researchers with insights into how students dealt with such conditions in the past. Thus, we contribute to understanding contemporary mental health issues and to preserving a valuable snapshot of mental wellbeing and adaptation, along with representative examples (see “Appendix 2”), during one of the most significant public health crises in modern history.

### Limitations

We acknowledge certain limitations in this paper. There’s a potential for sample bias in our combined sample from our own university environment and Prolific participants: The self-selected participant pool does not represent the exact demographic and psychographic structure of higher education. In addition, the two cohorts differed in recruitment procedures and eligibility criteria, which may limit their direct comparability. Further, differences in incentive modalities may slightly affect response style or length, but we argue our results and analyses still provide deep insights and value, as evident in the results overall. The combined input from both sources presents a compelling way to peek into the mental wellbeing landscape of modern higher education, and future work could look into how different sampling strategies and sources affect data overlap. In addition, although most participants were in early adulthood, the sample included a wide age range and students at bachelor’s, master’s, and doctoral levels, so this heterogeneity may limit the interpretability of our findings across different life and degree stages.

Although BERTopic generated satisfying results, there is an observed level of similarity or overlap between some of the topics. Some documents may fit in several topics. This overlap can be attributed to the inherent complexity and ambiguity present in natural language. BERTopic relies on semantic similarity, and while it excels in capturing general semantic patterns, it may struggle with distinguishing subtle differences in meaning. It is important to acknowledge that topic assignment is not a deterministic process, and the boundaries between topics may lack clear distinctions. Additionally, the limitations of BERTopic, including its reliance on pre-trained language representations and potential challenges in capturing nuanced semantic variations, should be considered when interpreting the results. Moreover, each response was assigned to a single topic, even though many responses contained multiple aspects, which may limit the granularity and accuracy of topic assignments. The manual merging of topics may also vary depending on the researcher’s interpretation, though the overall thematic patterns are expected to remain consistent. Formal topic stability or coherence metrics were not computed. Consequently, the identified themes should be interpreted as indicative rather than definitive. However, we argue this does not pose a significant concern as the topics were later examined manually. We also acknowledge that topic merging and labeling reflects the first author’s perspective and may vary if repeated by others.

Our study also employed non-probability sampling, with mixed recruitment sources and incentives (campus advertisements with raffles versus Prolific monetary compensation), which may have attracted participants with varying motivations. Additionally, the multilingual data processing and broad sample heterogeneity in age (18–70 years) and degree levels limit the overall generalisability of our findings. As stated earlier, these factors further suggest our findings should be interpreted as indicative patterns rather than universally representative of all higher education students’ mental wellbeing experiences. We argue that the broad sample and the resulting technology implications together make a meaningful contribution to the field of mental health, specifically in higher education.

Finally, given that data collection occurred, partially, during the COVID-19 pandemic, our findings may have limited timeliness. This also influences generalisability: The pandemic’s influence on participants’ mental and emotional states most certainly can affect results, and our findings should be interpreted as applicable primarily to this unique point in time. The timing constrains the direct one-to-one applicability of our results to typical higher education environments of today, and we see potential in future work to compare how the prevailing themes in data change as a function of distance from the pandemic and to assess the stability of these patterns across different contexts. Our current work, we posit, provides valuable insights into mental wellbeing during an unprecedented global crisis.

## Conclusion

In this work, we uncovered challenges in higher education, from depression to the repercussions of the COVID-19 pandemic. The challenges highlight the complex emotional and psychological issues that the current generation of students face. While these challenges are serious, it is also clear that the community is resourceful. They actively seek self-care methodologies, predominantly online, searching for methods that are peer-recommended, dependable, economical, and easily applicable in their lives. With a greater understanding of these issues, future technologies can be tailored more finely to resonate with the students’ realities, offering them avenues for self-improvement, growth, and coping.

Recent developments in LLMs suggest intriguing possibilities for supporting personalized self-care. Although our study did not evaluate AI-driven interventions, future work could investigate student-powered systems augmented by LLMs. Such applications would need rigorous safeguards, including strategies to mitigate misinformation, appropriate screening for mental health risk, and clear escalation protocols. We present LLMs as a promising avenue for future research rather than as conclusions supported by our current data.

## Data Availability

The data collected during this study are pseudonymised and are available for researchers upon request. However, the trained BERTopic models are publicly available at https://osf.io/yscrf/files/osfstorage?view_only=ff57a49539374d9 abb7ed8e67d672c7b.

## Appendices

### Appendix 1: Data protection disclaimer

#### Appendix 1.1: The controller

University of Oulu, Address: Pentti Kaiterankatu 1, 90570 Oulu, Finland

#### Appendix 1.2: Contact details for this research

Name: Simo Hosio

Address: University of Oulu, Pentti Kaiterankatu 1, 90570 Oulu

Email: simo.hosio@ouul.fi

#### Appendix 1.3: Purpose of the processing of personal data

The purpose of the processing of personal data is for scientific research. The study solicits contributions and assessments from members of the academic community regarding mental health self-care techniques.

The survey is conducted Answerbot3000 software, an online application owned by the Crowd Computing Research Group at the University of Oulu.

Participation in the study is voluntary, and subjects are informed in advance about matters related to participation in the study and the processing of the material. We also tell respondents the project home page where they can follow the progress of the study, and that they have the opportunity to ask the research team for more information about the study.

#### Appendix 1.4: Parties to the collaborative study and division of responsibilities

The survey is carried out by the Crowd Computing research group at the University of Oulu.

All requests related to this investigation (including requests for the exercise of the data subject’s rights referred to in Chapter III of the Data Protection Regulation) shall be addressed to the director responsible for the investigation:

Name: Simo Hosio

Email: simo.hosio@oulu.fi

#### Appendix 1.5: The director responsible for the research group running the study

Research group: Crowd Computing

Group Leader: Professor Simo Hosio

Email: simo.hosio@oulu.fi

#### Appendix 1.6: Name, nature and duration of the study

Title of the study: Crowdsourcing mental health self-care solutions among university students

One-time study

Duration of the investigation (how long the personal data will be processed): 22.05.2020; We will collect data until thus far unspecified time, depending on the success of data collection

#### Appendix 1.7: Legal basis for the processing of personal data

EU General Data Protection Regulation, Article 6 (1) and Section 4 of the Data Protection Act:

- Consent of the data subject
- Compliance with the data subject’s legal obligation

Instruments:

- Scientific or historical or statistical research

#### Appendix 1.8: Sensitive personal data (data belonging to specific categories of personal data and criminal data)

The study does not deal with sensitive personal data.

The investigation does not deal with information on a criminal conviction or offence.

#### Appendix 1.9: Information content of the research register

The personal data collected are gender, age, and academic faculty. We also ask for email in a separate form if the partipant opts to join in the prize draw, hosted on a different form service offered by the University of Oulu. In addition to background information, the survey asks users to assess existing mental health self-care techniques and also to contribute new ones. None of this is personally identifiable data.

#### Appendix 1.10: Sources of personal data

Participants are approached through their associated Student Guilds. We also extensively use social media to promote the study. The personal data we have about respondents is provided by the respondents themselves in the questionnaire (e.g. gender and age).

#### Appendix 1.11: Transfer or disclosure of personal data outside the research team

Data will not be transferred outside the study group during the study. The anonymized material will be stored at the AnswerBot3000 database (ab3000.net).

#### Appendix 1.12: Transfer of personal data outside the EU/EEA

Data collected is not transferred to a third country or to any organization outside the EU or the EEA

#### Appendix 1.13: Automated decision making

No automatic decisions are made.

#### Appendix 1.14: Registry security principles

Privacy is taken care of. Respondents’ email addresses (only given for the purposes of the prize draw) and responses are not combined at any stage of the survey (and in fact, cannot be combined as they are collected separately without storing e.g. timestamps, IP addresses or any such information that could be theoretically used to connect the data).

Processing of direct identification data: Direct identification data is not collected.

Data protection in data transfers: Data transfer encryption: The material is handled anonymously at all stages. In this study, responses are not associated with data tags at any stage.

Data encryption: The material is stored behind strong credentials (password-protected). Further, the material is only available to project leader.

#### Appendix 1.15: Processing of personal data after the end of the investigation

The research register is archived without identification data

Where the material will be archived and for how long:

After the implementation of the study in 2020, the survey material will be archived for research and development purposes.

#### Appendix 1.16: Data subject’s rights and possible limitation

We will offer the parcipants a chance to delete their data through a randomly assigned identifier that they will have to store during the data collection period.

Subject to data protection law, the data subject has:

- *Right to inspect data (right to access personal data)*
  The data subject has the right to know whether or not his or her personal data are being processed and what personal data about him or her has been stored.
- *Right to rectification*
  The data subject has the right to request that incorrect, inaccurate or incomplete personal data concerning him or her be corrected or supplemented without undue delay. In addition, the person has the right to demand that unnecessary personal data be deleted.
- *Right to delete data*
  In exceptional cases, the data subject has the right to have his or her personal data completely deleted from the controller’s registers (right to be forgotten).
- *Right to restrict processing*
  The data subject has the right, in certain situations, to request that the processing of his or her personal data be limited until his or her data has been duly checked and corrected or supplemented.
- *Of opposition*
  In certain situations, a person has the right at any time, based on his or her personal, special situation, to oppose the processing of his or her personal data.
- *The right to transfer data from one system to another*
  In certain situations, the data subject has the right to obtain personal data concerning him which he has provided to the controller, in a structured, commonly used and machine-readable form, and the right to transfer the data to another controller.
- *Reasons for restriction*
  During the data collection, the respondent has the right not to participate in the survey. Due to the unanonymized data collection procedure we use, we are unable to identify a respondent’s data without sufficient information about the particulars of the data since their responses will be stored in the system anonymously. Therefore, the data of an individual defendant cannot be ascertained from him or her at a later stage.
- *Right to appeal to the supervisory authority*
  The data subject has the right to lodge a complaint, in particular with the supervisory authority at the University of Oulu, if he or she considers that the processing of personal data violates the general EU data protection regulation (EU) 2016/679. The data subject also has the right to use administrative appeals as well as other legal remedies.

*Contact:*

Professor Simo Hosio

Email: simo.hosio@oulu.fi

Requests for the exercise of the data subject’s rights shall follow the data request process of the controller.

### Appendix 2: Topic merging and sample documents

The following Tables 4, 5, 6 and 7 present the topics generated by BERTopic. Each table displays the initial BERTopic-generated topics and their IDs under the ‘Original Topic‘ column, along with their manually grouped and labeled counterparts in the ‘Merged Topic‘ column for a specific question. The tables also report the number of documents belonging to each original topic and include a sample of documents for each topic.

**Table 4:** BERTopic-generated topics (Original Topic) and their manually merged and labeled counterparts (Merged Topic) for Questions 1: ‘Where would one find self-care techniques’

Merged topic	Original topic	Documents count	Sample documents
Outliers	− 1	1	“I would try to talk to friends and family about it to know if they know about anything about it, and then start searching on the internet too.”, “Google, friends and family, social media”, “Google mental health or personal development. Go read self improvement and growth subreddits on Reddit”
Google	0	180	“Search about mental health care in internet.”, “I’d look for other people’s mental health self-care techniques and go from there.”, “I would probably Google mental health self care techniques and go from there.”]
	1	92	“I would use google to find those techniques”, “I would likely start searching online for techniques, maybe ask family.”, “I would google different options and tips”
	4	62	“InternetGoogle ”, “Internet and google.”, “From the internet, google”
	6	32	“I would google it.”, “I’d google it”, “I would google it. See which ones sound like bogus and which ones don’t”
Internet	5	48	“Internet. I would search there first.”, “I would (and have) search on the internet.”, “I will search the internet first”
	9	28	“Internet”, “From the internet”, “From internet”
Youtube	2	72	“Maybe Youtube”, “Youtube”, “From YouTube”, “Google, youtube”
Exercise/Meditation	3	63	“I would search for meditation apps and self help books. I would start going for a long walks and try to speak about it.”, “Mood cafe would be somewhere i would start.”, “Going on more walks outside and getting a more consistent sleep schedule”
Friends/Family	7	32	“In the internet or asking friends about this.”, “From internet or asking friends.”, “Google it and asking friends”
	14	29	“I would probably search online for ideas, or ask close friends.”, “Probably searching online. I would also go to my friends and ask them.”, “I would search online or ask friends for their help”
Mental health websites	11	22	“I would go to trusted internet page related to health, i.e. Mielenterveystalo.”, “Student health care website, municipality health care website, just google things.”, “I would Google the Internet and look for information in the student health website”
	12	23	“Search from yths website.”, “YTHS.”, “terveyskyla.fi mielenterveystalo”
Experts	10	18	“I would seek the help of a counsellor or therapist.”, “I would go to school doctor and tell my problems. I wish that doctor could help me to go psychologist or somebody else”
	13	20	“Books.”, “I’d do research on the topic: read articles and books.”, “I would read some book about the subject”
Solutions within	8	26	“U should start asking yourself why are you feeling a certain way.”, “I feel like thats more of a thing that finds you, a thing you find joy in and do in order to feel better about yourself.”, “Maybe I’d just think first—what makes me feel happy, relaxed or good? And then do those things. Like example walking in nature”

The table also shows the number of documents belonging to each original topic and example documents

**Table 5:** BERTopic-generated topics (Original Topic) and their manually merged and labeled counterparts (Merged Topic) for Questions 2: ‘What would affect choosing a self-care technique’

Merged topic	Original topic	Documents count	Sample documents
Outliers	− 1	5	“I value scientific approach to mental health problems. Even though I believe there are some more down-to-earth solutions and techniques to mental health care, they can not solve the deepest problems one might have. When considering if something is scientific or not, I research the author of the book or the “founder” of the technique from Internet and then evaluate its competence. And of course if I have had recommendations from my family or friends, it would affect my choice in a way or another, but I still want to research the technique on my own”
Source reliability	1	199	“Personally I have tried several techniques already. I find it important that the method is backed up by scientific evidence. My choice is also affected by other people’s experiences on trying the same method.”, “The credibility of the source; what my friends have tried if I ask them about different things or if something during my google search is a technique I know they have tried”
Ease of use	0	179	“It’s easy.”, “It is easy to do.”, “Easy to do”
Personal preference	3	84	“If someone has had a bad experience with a certain self-care technique.”, “I would want to try self-care techniques that are doable and that are indeed self-care techinques and not excuses to self-indulge”
	4	58	“I might try Yoga as an alternative and laziness could set in which could affect my choices.”, “i think I learn and grow more through mental activity so i would focus on that.”, “I’d leave out techniques that sounds too weird or makes me think it’s foolish”
	10	18	“I will think about result and if there is any effects.”, “I would consider how it fits with my personality, also I would value it if it doesn’t require a great amount of time and if it can be done every day.”, “How it would fit my lifestyle”
Affordability	2	68	“Easy access, price, good reviews and proven results.”, “The price.”, “Price and time”
User reviews	6	38	“Other people’s opinions in the internet would affect my choice.”, “My environment will affect my choice and the people around me.”, “My choice would be affected by who recoomends a certain procedure and how much better they felt”
	9	21	“Other peoples comments and opinions.”, “Reviews.”, “reviews by different users, user experiences”
Prior experience	5	44	“Experiences.”, “The experiences of others.”, “I prefer authentic, personal stories and experiences over professional advice”
Time	7	35	“How much time would it take to do, how much effort would I need to put into it.”, “Time that it takes.”, “Vaatiiko tekniikka paljon aikaa ja voiko sitä tehdä monessa tilanteessa vai pelkästään kotona tai yksin. Tuntuuko tekniikka luontevalta itselle”
Recommendations	8	25	“Friends’ recommendations and probably strangers recommendations on jodel etc.”, “If I have heard s friend say good things about a specific resource I would be inclined to try that thing.”, “Good recommendations and feed back from many diffrrent people. Something that has good and thourough explenations and intructions”

The table also shows the number of documents belonging to each original topic and example documents

**Table 6:** BERTopic-generated topics (Original Topic) and their manually merged and labeled counterparts (Merged Topic) for Questions 3: ‘Identifying troubling things in life’

Merged topic	Original topic	Documents count	Sample documents
Outliers	− 1	7	“Relationship problems, family, studies and work.”, “Relationship stress. Stress from work. Anxiety. Suicidal thoughts.”, “School, stress, relationships”
Stress	5	51	“Stressing about every unsure or unstable situation (financial situation, health issues, not being independent).”, “Illness of a family member. Work stress.”, “Having too little sleep, worrying about the health of loved ones”
	10	59	“Stress is a big factor and fear of the future.”, “Feeling enough for my parents, setting realistic life goals and managing my expectations regarding them, dealing with failure”
	13	45	“School, stress, relationships.”, “The college and some relationships.”, “Stress about school or look-related thinhgs”
Study	0	86	“Working (= doing my doctoral studies) too much, having too many projects at the University, having too tight schedules, feeling insufficient, seeing relationships deteriorating because of not having enough time or energy to spend time with them, being afraid of losing friends because of work, not having enough time to exercise, sleep and recover, losing the control of my life.”, “School, work, money, stress”
Depression	1	83	“Depression and anxiety.”, “depression, anxiety.”, “The depression that hits when failing on something you shouldn’t”
Loneliness	2	75	“Loneliness, decisions, abuse.”, “Loneliness, future.”, “Breakups in longer relationships”
Childhood trauma	3	55	“Childhood trauma from family, bullying in school, ending relationships, social media, physical accidents, escaping feelings in doing a lot instead of feeling them.”, “Getting bullied in school, my parents being unemployed and not having money etc, other family problems...”, “Some of my childhood memories”
Financial matters	7	54	“Money, work.”, “Work, money, relationship, studies, family and sometimes life in general.”, “Money, studies, work”
Future uncertainty	6	46	“Future.”, “Thinking about the future.”, “my career and my future as well as keeping relationships important to me”
Low self-esteem	4	44	“Anxiety, stress, self-esteem.”, “Body image issues, no self-worth.”, “Body image issues, no self-worth”
Relationships	9	38	“For sure the romantic relationships. The searching for professional experience as well. And recently, the uncertainty of our political environment.”, “relationships with some people.”, “Relationships”
Death of loved ones	8	34	“The death of loved ones.”, “losing someone i loved to death.”, “Losing of someone I love, traumatic events”
Emotional issues	14	30	“Stress, not feeling like I’m enough, physical health.”, “The dark during fall and winter.”, “Not knowing if I could continue. If things would every get better, this affected my everyday life”
Alcohol	12	25	“My past history with substance abuse.”, “the alcohol problems of someone very close to me, financial issues.”, “Family members alcoholism, my health issues”
Family problems	15	21	“My relationship with my father. Not beeing in relationship with anyone.”, “Raising two young children is a strain mentally.”, “Relationships, my fathers approving of me, future carreer decisions”
Eating disorder	11	21	“Eating disorder. Anxiety and depression.”, “Traumas, eating disorder.”, “Body image, anxiety, eating disorder”

The table also shows the number of documents belonging to each original topic and example documents

**Table 7:** BERTopic-generated topics (Original Topic) and their manually merged and labeled counterparts (Merged Topic) for Questions 4: ‘What could help’

Merged topic	Original topic	Documents count	Sample documents
Outliers	− 1	4	“I have no cure, unfortunately.”, “to disconnect.”, “A higher IQ.”, “Perhe, ystävät, mieluisa tekeminen”
Social support and connection	1	47	“They can help me by listening to me.”, “Someone could help me talking and giving advice with immediate effect.”, “Showing that u understand and support the person is a good start... Talk and explore the problem”
	4	26	“Having a good support system round me helps. Knowing I have got close people I can open up to.”, “Having more support from family, having time to myself.”, “Friends and family support, professional councelling”
	14	24	“It could help to get guidance on what things to drop when I have too much to do.”, “Speaking with a professional, psychotherapy, mercy to oneself, doing pleasant things in life (hobbies etc.).”, “Therapy and counselling, guidance”
	18	12	“Journaling, talking to friends or finding some sort of support groups online, listening to music and doing what I like doing.”, “Time from happenings, making art and praying God and talking whit trusted people or professionals.”, “Hanging with loved ones, talking about how I feel, walking, swimming, yoga, eating good food with someone I like, art”
	19	13	“Staying in contact with friends and family, not completely isolating oneself.”, “Talking with people. Trying to see yourself from new point of view. Universal Basic Income.”, “I guess finding ways to connect with people or to be comfortable with being alone”
	22	12	“Being around other people, face to face discussions, someone to cheer me.”, “Talking to someone about my experiences, preferably to a therapist or other professional.”, “I think the most helpful thing would be that the people around you understand the situation and pressure you’re under. Then maybe they can help you by demanding you take time off from all those things and they could also demand that you do something fun together”
	23	11	“Trying to socialize more.”, “Open communication with my friends and relaxing once in a while.”, “Talking to my friends and peers/finding like minded people in similar situations”
	27	10	“Discussing with people.”, “Discussion.”, “Talking, guides how to contact or start discussion after a fight, how to find the right words”
	37	9	“Talking to people I trust.”, “Having a discussion with someone in the same situation, or the people who I worry about.”, “Talking to someone about my concerns and remembering to take it easy and not too seriously”
	38	5	“Listen to stories from someone who had similar experiences and was able to overcome that obstacles of the past, an was able to move on stronger.”, “I needed to realize that even though we all need supportive people around us, it is not enough. Only you can change your own life and work for your achievements. Victimhood is nothing but an excuse that allows you to fail again and again. You need to move, even little steps are better than no steps at all.”, “ensimmäiseen ystävien tuki ja rohkaisu ottamaan asian puheeksi kotona. epäonnistumisia voisi myös helpottaa ja kompensoida kokemus onnistumisesta”
	39	20	“Talking with friends or family.”, “Speaking to other family members.”, “People to talk to and reaching out for help”
	46	5	“Perhaps more people to hang out with and talk to about the situation. I know sports aren’t really a big deal but, letting everyone down does hurt. At some stage, I wish I could find a new circle and forget the people who had negatively affected me. However it is just hard when such circle is too close in proximity and you have to deal with them everytime”
	49	6	“Practicing social skills and dealing with my past experiences.”, “open conversation with someone I trust, social interactions, scheduling my studying and free-time, doing something good for my well-being and accepting I can’t do everything, finding ways to solve the value conflicts I have. Taking time to relax and self-care and not feel bad about it”
Therapy and professional help	13	15	“I am in therapy right now, which I have considered helping.”, “I have a medication and I go to therapy regularly.”, “Professional help, and I’m already in therapy”
	16	18	“I think talking with professional could help, but it is hart to find professional and it might be expensive.”, “A healthcare professional who could be reached on a low threshold without long waiting times.”, “Talking to a professional about them”
	17	12	“Therapy, group support, being able to tell all about it to someone with similar background and experience, outside help/social work from the city of Oulu.”
	20	13	“I think that the profesional help is allways best, but I survived without it.”, “I have heard that congitive behavioral therapy helps many people who suffer from OCD”
	21	12	“Therapy and ice cream.”, “Therapy with a professional who is specialized on the subject.”, “Therapy and different methods and mechanics I learned there”
	30	7	“Actual treatment for my eating disorder and more dialectical behavioural therapy and/or mentalisation.”, “Treatment balance about the depression I have and taking up psychotherapy. Also proper sheduling”
	34	11	“Drugs (only sort of kidding). Therapy.”, “idk I guess therapy or meds.”, “Therapy, medication, friends, family”
	42	10	“Therapy or therapy based exercises done regularly.”, “A therapist, definitely. Maybe some mental exercises.”, “I do yoga and run. Also I have talked with therapist about my traumas”
	43	6	“I’d think different kinds of therapy or counseling would be awesome, and maybe some programmes where you don’t need another person to guide you.”, “Professional help (I.e. counseling, psychotherapy, medicine), support from community (friends, family)”
	45	7	“Informing people about mental health, talking to professionals, being able to have access to enough help for free.”, “maybe talk to a specialist to help me”
	50	6	“Speaking to friends helps, also therapy would probably help.”, “I think in most of cases a good friend or psychologist could help you to pass through this situation.”, “My psychologist is helping me a lot and I trust her”
	51	12	“Go see a therapist.”, “probably a psychologist.”, “Access to therapists and other professional help”
Stress management and relaxation techniques	3	26	“Someone could help me if he/she showed me stress management techniques.”, “something to help with the anxiety of dealing with new situations and environments that does NOT involve CBT.”, “Maybe better-studying techniques and someone to guide me through the feelings of anxiety”
	5	23	“Meditation, mindfulness, yoga, and therapy.”, “therapy, meditation.”, “Meditation”
	7	25	“As I mentioned already yoga and fresh air always helps to reduce stress and maintain mental health.”, “Different calming things help (for example calm music).”, “I think I would benefit for example different stress relieve techniques”
	15	13	“Try to let go of being in control all the time.”, “Accepting that nobodys perfect. Thinking that the way you acted at the moment was with the best knowledge that you had at the moment and you tried your vest at the situation what was then”
	40	12	“ITechiques to relax. Someone to talk to.”, “Simple routines i could follow, discussions.”, “i think it would be good to learn how to relax and not to think too much”
	47	6	“Relaxing and taking easy in life.”, “I need to learn some relaxing methods and have more leisure time.”, “In my case, rest. Rest, rest and rest. Doing things that I enjoy or want to do at that moment and not stressing about undone tasks. Christmas break was very needed and the quality time with family”
	52	10	“Talk to other people about these things and mindfullness practises.”, “Hearing others experiences, talking to friends and asking for support, guided meditation.”, “I usually try talking about them with a trusted friend or loved one. I also try some other techniques like breathing exercises and meditation”
	53	4	“Talking to people I trust, such as friends or family. Distracting myself and trying to cope instead of focusing on the negative events and emotions. Professional help, such as counselor and psychoterapists.”, “Self-confidence”
Positive mindset	0	68	“Try to live life in current day rather than thinking about the future.”, “I think teaching me how to recognize and defuse how I’m feeling would help.”, “Drugs and maybe a change in mindset”
	11	20	“Not pushing me to do things in a hurry; trying to help me organise and structure a work effective schedule.”, “I think all I need is some discipline and time to reflect on things outside of school”
	28	9	“Talking more openly with my loved ones about things and major decisions.”, “being able to openly express myself without negative repercussions.”, “Talk more open about all relationships. Act mote kindly to myself”
	36	8	“To find what gets you motivated, or to motivate yourself.”, “Trying to stay active and find inspirational things to do no matter what”
Self-reflection	10	17	“I don’t know exactly but I think sometimes you need to let everything flow and times would solve everything.”, “I have to find a passion that gives me pleasure and maybe in the future it will result in good work”
	24	18	“The best thing is to get me up from the bed if I’m ever stuck in there. And get moving and doing things.”, “The only thing that is really helping me is to take some time for myself, to dedicate something to myself. Doing what I really like, even alone, as long as it is something I like. Something about my passions”
	25	7	“Nothing, really.”, “Nothing really.”, “Idk man”
	32	9	“Just opening up to someone who I trust about the things from my past that I keep dwelling on.”, “I think confidince building would be a good starting place and taking it from there”
	41	7	“I’ve had times where I took somebody as a good example or times that hanging out with friends or going for a walk made me more productive.”, “Talk it out with a specialist or writing a journal to clear up my mind”
	44	7	“Talking to other people, realizing everyone has doubts.”, “Previous experiences shared. Looking forward and trying to feel positive.”, “I could improve my body image by exercising or by not comparing myself to others”
Financial support and stability	6	19	“Getting reassurance of job security and more rewarding activities in life.”, “Working as hards as possible so I don’t have to worry.”, “Career change or new job tasks. Or new initiatives to increase job satisfaction. For exemple, I have a lot to improve in time management”
	9	15	“Give me money and i will be fine.”, “I need funding for a business idea that I’ve been having for some time. Also a better paying job would assist a lot.”, “I think the only thing that would help me is if I didn’t have to worry about money”
	26	7	“Financially: I am actively searching for a job so with that, I don’t know how someone else can actually help. I know this is a major issue for most of the foreign people in Finland that they don’t usually find a job after their studies. Health: In this case, I am grateful I have access to student health service. However, there is a long queue for an appointment and sometimes I become worried to wait for an appointment while I m experiencing unpleasant symptoms. I know it will get better once I have a full time job where I am going to have a work healthy insurance. Independent: I don’t feel settled in Finland since I couldn’t find a job yet and living in the student apartment is for a limitated time. A plan B would be to find a job in my domain in another foreign country and trying to have a stable life there but at the moment I am considering settling in Finland. So overall, my stressing situation is resumed to having a job in my domain which will take time to find”
	48	5	“Therapy probably, but that’s expensive. Otherwise just living well.”, “Therapy, for sure. Also meds, but that things cost money which i don’t really have at the moment.”, “My medical condition that has affected my mental health is called Bromhidrosis and the thing that could help with this issue would be money so I could pay a good dermatologist to treat my problem”
Understanding and awareness	2	30	“No idea.”, “I have no idea.”, “I really have no idea about that”
	29	7	“No help, it’s a personal thing that needs to be resolved internally.”, “Talk about the issues with experts that are ready listen. Get through you worries so getting done with the work to relieve the stress its causing for example”
Physical exercise and healthy lifestyle	12	13	“Better diet, better sleep pattern.”, “Find a balance in life to get enough sleep, learning to meditate and relax before sleep. Help loved ones so that they stay healthy.”, “A healthy sleep routine and time management skills help. Also letting loose and hanging out with friends”
	33	13	“Knowing that everyone is healthy is the biggest one. Having some events or school at campus (risk free ofc).”, “My horse helps”
Education and information	31	13	“Getting a tutor would help me with my studies and i would perform better.”, “Studying less, finding new ways to study.”, “Something could help me by facilitating ways to ameliorate this condition”
	35	11	“I could have requested help at a special advice center of the university for psychological well-being.”, “I would especially need help from my professors taking care of thesis writers.”, “Society could definitely do more... something for young adults! Or university could help”
Time management and organization	8	16	“In stress it would help to find perfect balance with studying and freetime. I should also be more merciful for myself.”, “Techniques for planning workloads ahead have helped before and would probably help now. Better guiding for my studies would relief stress from uncertainties and/or progressing my studies”

The table also shows the number of documents belonging to each original topic and example documents

### Appendix 3: Prolific participants demographics

See Tables 8 and 9.

**Table 8:** Country distribution of prolific participants

Country	Count	Percent (%)
United States	53	18.2
South Africa	45	15.5
Portugal	33	11.3
United Kingdom	30	10.3
Mexico	25	8.6
Poland	23	7.9
Italy	22	7.6
Germany	15	5.2
Greece	11	3.8
Spain	7	2.4
Chile	6	2.1
Hungary	4	1.4
Slovenia	4	1.4
Austria	3	1.0
Australia	2	0.7
Canada	1	0.3
Estonia	1	0.3
Netherlands	1	0.3
Denmark	1	0.3
Korea	1	0.3
Israel	1	0.3
Belgium	1	0.3
Sweden	1	0.3

**Table 9:** Language distribution of prolific participants

Language	Count	Percent (%)
English	126	43.3
Spanish	37	12.7
Portuguese	33	11.3
Polish	23	7.9
Italian	21	7.2
German	17	5.8
Greek	11	3.8
Afrikaans	3	1.0
Hungarian	3	1.0
Slovenian	2	0.7
Arabic	2	0.7
Dutch	2	0.7
Punjabi	2	0.7
Tamil	1	0.3
Estonian	1	0.3
Chinese	1	0.3
French	1	0.3
Other	1	0.3
Ukrainian	1	0.3
Russian	1	0.3
Swedish	1	0.3
Bengali	1	0.3

### Appendix 4: Sankey diagram: top 3 flows per source topic

See Tables 10, 11 and 12.

**Table 10:** Top 3 flows per source topic from Question 1 to 2

Source topic	Target topic	Count (%)
Google	Source reliability	111 (30.4)
	Personal preference	76 (20.8)
	Ease of use	75 (20.5)
Exercise/meditation	Personal preference	26 (41.3)
	Ease of use	17 (27.0)
	Source reliability	7 (11.1)
Internet	Ease of use	28 (37.3)
	Source reliability	18 (24.0)
	Personal preference	8 (10.7)
YouTube	Source reliability	16 (22.2)
	Ease of use	14 (19.4)
	User reviews	12 (16.7)
Experts	Ease of use	9 (24.3)
	Source reliability	8 (21.6)
	Personal preference	7 (18.9)
Friends/family	Source reliability	17 (27.9)
	Ease of use	14 (23.0)
	Personal preference	10 (16.4)
Mental health websites	Source reliability	14 (31.1)
	Ease of use	11 (24.4)
	Personal preference	11 (24.4)
Solutions within	Personal preference	11 (42.3)
	Prior experience	3 (11.5)
	Ease of use	3 (11.5)

**Table 11:** Top 3 flows per source topic from Question 2 to 3

Source topic	Target topic	Count (%)
Ease of use	Stress	39 (21.9)
	Study	23 (12.9)
	Depression	20 (11.2)
Source reliability	Stress	31 (15.6)
	Depression	28 (14.1)
	Study	20 (10.1)
Personal preference	Stress	32 (20.6)
	Loneliness	21 (13.5)
	Study	19 (12.3)
Time	Stress	10 (29.4)
	Loneliness	7 (20.6)
	Study	4 (11.8)
User reviews	Stress	17 (29.3)
	Death of loved ones	8 (13.8)
	Future uncertainty	5 (8.6)
Prior experience	Stress	12 (27.3)
	Depression	7 (15.9)
	Low self-esteem	5 (11.4)
Affordability	Financial matters	11 (16.2)
	Stress	10 (14.7)
	Relationships	9 (13.2)
Recommendations	Study	5 (20.0)
	Future uncertainty	4 (16.0)
	Depression	4 (16.0)

**Table 12:** Top 3 flows per source topic from question 3 to 4

Source topic	Target topic	Count (%)
Eating disorder	Therapy and professional help	8 (38.1)
	Social support and connection	4 (19.0)
	Positive mindset	4 (19.0)
Loneliness	Social support and connection	31 (41.3)
	Positive mindset	11 (14.7)
	Therapy and professional help	8 (10.7)
Stress	Social support and connection	34 (22.4)
	Stress management and relaxation techniques	29 (19.1)
	Positive mindset	23 (15.1)
Childhood trauma	Therapy and professional help	16 (29.6)
	Social support and connection	13 (24.1)
	Positive mindset	7 (13.0)
Depression	Therapy and professional help	24 (29.3)
	Stress management and relaxation techniques	23 (28.0)
	Positive mindset	12 (14.6)
Alcohol	Therapy and professional help	7 (28.0)
	Social support and connection	6 (24.0)
	Stress management and relaxation techniques	3 (12.0)
Study	Social support and connection	23 (27.1)
	Positive mindset	12 (14.1)
	Stress management and relaxation techniques	11 (12.9)
Low self-esteem	Social support and connection	11 (26.8)
	Self-reflection	7 (17.1)
	Positive mindset	7 (17.1)
Emotional issues	Social support and connection	8 (26.7)
	Stress management and relaxation techniques	6 (20.0)
	Therapy and professional help	4 (13.3)
Death of loved ones	Social support and connection	17 (50.0)
	Therapy and professional help	5 (14.7)
	Positive mindset	4 (11.8)
Financial matters	Social support and connection	13 (24.1)
	Financial support and stability	10 (18.5)
	Stress management and relaxation techniques	9 (16.7)
Family problems	Social support and connection	10 (50.0)
	Financial support and stability	2 (10.0)
	Understanding and awareness	2 (10.0)
Relationships	Social support and connection	12 (31.6)
	Self-reflection	9 (23.7)
	Stress management and relaxation techniques	5 (13.2)
Future uncertainty	Stress management and relaxation techniques	11 (24.4)
	Positive mindset	9 (20.0)
	Self-reflection	7 (15.6)

## Footnotes

1 https://osf.io/yscrf/files/osfstorage?view_only=ff57a49539374d9abb7ed8e67d672c7b.
